# Design, synthesis, conformational and molecular docking study of some novel acyl hydrazone based molecular hybrids as antimalarial and antimicrobial agents

**DOI:** 10.1186/s13065-017-0344-7

**Published:** 2017-11-14

**Authors:** Parvin Kumar, Kulbir Kadyan, Meenakshi Duhan, Jayant Sindhu, Vineeta Singh, Baljeet Singh Saharan

**Affiliations:** 1 Department of Chemistry, Kurukshetra University, Kurukshetra, 136119 India; 2 S D (PG) College, Panipat, 132103 India; 3 National Institute of Malaria Research, Dwarka, New Delhi 110077 India; 4 Department of Microbiology, Kurukshetra University, Kurukshetra, 136119 India

**Keywords:** Antimalarial, DHP, Pyrazole, Conformational studies, *Plasmodium falciparum*, Falcipain-2, Antimicrobial, Antifungal

## Abstract

**Background:**

Acyl hydrazones are an important class of heterocyclic compounds promising pharmacological characteristics. Malaria is a life-threatening mosquito-borne blood disease caused by a plasmodium parasite. In some places, malaria can be treated and controlled with early diagnosis. However, some countries lack the resources to do this effectively.

**Results:**

The present work involves the design and synthesis of some novel acyl hydrazone based molecular hybrids of 1,4-dihydropyridine and pyrazole (**5a**–**g**). These molecular hybrids were synthesised by condensation of 1,4-dihydropyridin-4-yl-phenoxyacetohydrazides with differently substituted pyrazole carbaldehyde. The final compound (**5**) showed two conformations (the major, *E*, *s*-*cis* and the minor, *E*, *s*-*trans*) as revealed by NMR spectral data and further supported by the energy calculations (MOPAC2016 using PM7 method). All the synthesised compounds were screened for their in vitro antimalarial activities against chloroquine-sensitive malaria parasite *Plasmodium falciparum* (3D7) and antimicrobial activity against Gram positive bacteria i.e. *Bacillus cereus*, Gram negative bacteria i.e. *Escherichia coli* and antifungal activity against one fungus i.e.* Aspergillus niger*. All these compounds were found more potent than chloroquine and clotrimazole, the standard drugs.

**Conclusions:**

In vitro antiplasmodial IC_50_ value of the most potent compound **5d** was found to be 4.40 nM which is even less than all the three reference drugs chloroquine (18.7 nM), pyrimethamine (11 nM) and artimisinin (6 nM). In silico binding study of compound **5d** with plasmodial cysteine protease falcipain-2 indicated the inhibition of falcipain-2 as the probable reason for the antimalarial potency of compound **5d**. All the compounds had shown good to excellent antimicrobial and antifungal activities. 
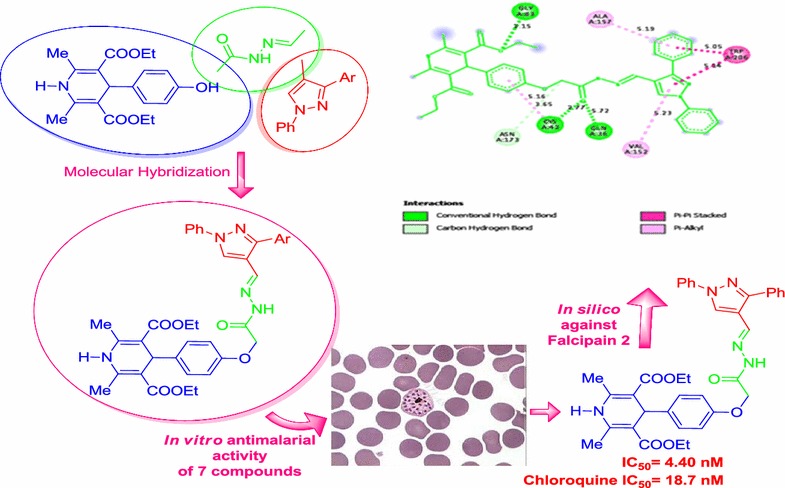

**Electronic supplementary material:**

The online version of this article (10.1186/s13065-017-0344-7) contains supplementary material, which is available to authorized users.

## Background

Malaria is a public health distress in countries in which this disease is prevalent. 50% of the world population is at risk of contacting the disease. Approximately one million people die annually owing to *Plasmodium falciparum* malarial infections, the majority of them are young children and pregnant women [1]. Several organised efforts to control the transmission and eradicate the disease have been made through history [2]. Complex life cycle, disease spreading through a mosquito vector, resistance to insecticides and a rapidly growing resistance to malarial parasite to the available drugs are the major reasons behind malaria proliferation [3–5]. The parasite is developing resistance against drugs, such as antifoliates and chloroquine, by random mutation [6]. Although five species of *Plasmodium* family of protozoan parasites can infect humans to cause malaria, *P. falciparum* and *P. vivax* are responsible for almost all malaria-related deaths.

Molecular hybridization as a drug discovery strategy involves the rational design of new chemical entities by the fusion (usually via a covalent linker) of two drugs, both active compounds and/or pharmacophoric units recognized and derived from known bioactive molecules [7–10]. The selection of the two principles in the dual drug is usually based on their observed synergistic pharmacological activities to enable the identification of highly active novel chemical entities.

**Fig. 1:**
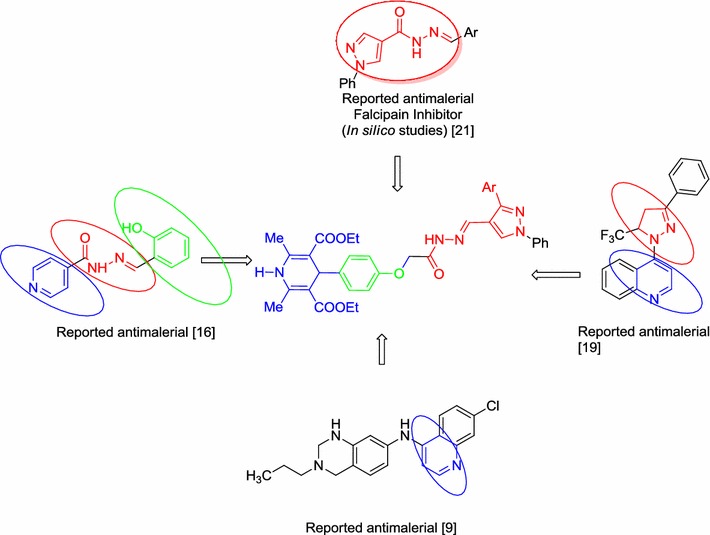
Drug designing by molecular hybridisation approach for the synthesis of new molecular hybrids

Pyrazole represents a class of heterocyclic compounds which exhibits significant biological properties such as antimalarial [11–13], antispasmodic [14], anti-inflammatory [15], antibacterial [16], analgesic [17], antihyperglycemic [18, 19], antineoplastic [20], antidepressive activities [21]. Similarly, pyridine ring has also been proved to be important scaffold as it has been present in various peptidomimetic and non-peptide falcipain inhibitors [22]. Virtual screening has also witnessed the importance of acyl hydrazones for the synthesis of non-peptide based falcipain inhibitors [23]. Therefore here in this study, we have decided to construct the molecular hybrids based on 1,4-DHP and pyrazole moieties using acyl hydrazone linkage which may possibly circumvent the antiplasmodial drug resistance (Fig. 1).

## Results and discussion

### Synthesis

**Scheme 1:**
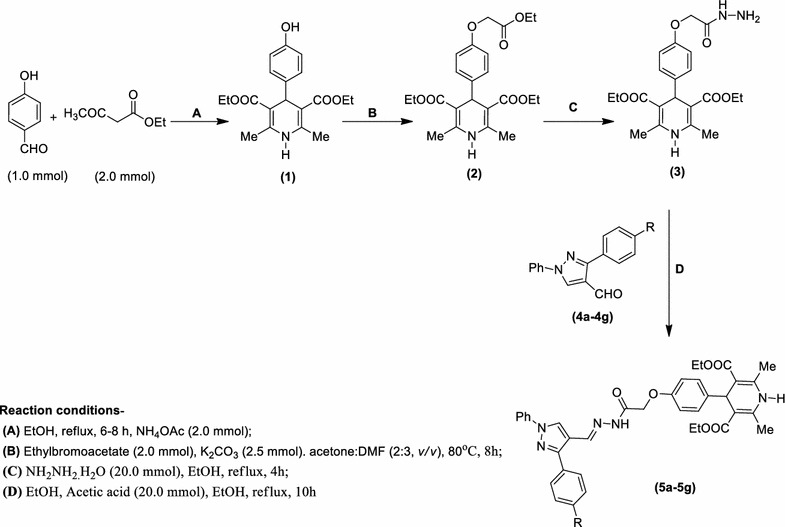
Synthesis of diethyl 4-(4-(((3-aryl-1-phenyl-1*H*-pyrazol-4-yl)methylene aminocarbamoyl)methoxy)phenyl)-1,4-dihydro-2,6-dimethylpyridine-3,5-dicarboxylate (**5a**–**g**)

The compound **5**(**a**–**g**) under investigation was synthesised (Scheme 1) in a 4-step process commencing from a three-component reaction [9] of ethylacetoacetate (2.00 mmol), 4-hydroxybenzaldehyde (1.00 mmol) and ammonium acetate (2.00 mmol) to obtain diethyl 1,4-dihydro-4-(4-hydroxyphenyl)-2,6-dimethylpyridine-3,5-dicarboxylate (**1**) which was subsequently converted to diethyl 4-(4-((ethoxycarbonyl)methoxy)phenyl)-1,4-dihydro-2,6-dimethylpyridine-3,5-dicarboxylate (**2**) by alkylation with ethyl bromoacetate. This DHP-based ester **2** was then reacted with hydrazine hydrate (20.00 mmol) to get 2-(4-(3,5-bis(ethoxycarbonyl)-2,6-dimethyl-1,4-dihydropyridin-4-yl)phenoxy)acetic acid hydrazide (**3**) which was condensed with 3-aryl-1-phenyl-1*H*-pyrazole-4-carbaldehyde (**4**) (1.00 mmol) using a catalytic amount of acetic acid in ethanol under reflux condition for 10 h to furnish 4-(4-(((3-aryl-1-phenyl-1*H*-pyrazol-4-yl)methyleneaminocarbamoyl)methoxy)phenyl)-1,4-dihydro-2,6-dimethylpyridine-3,5-dicarboxylate **5**(**a**–**g**) (92–98%) (Scheme 1; Table 1). The progress of the reaction in all cases was monitored by TLC examination using petroleum ether:ethyl acetate.

**Table 1:** Synthesis of diethyl 4-(4-(((3-aryl-1-phenyl-1*H*-pyrazol-4-yl)methyleneamino carbamoyl)methoxy)phenyl)-1,4-dihydro-2,6-dimethylpyridine-3,5-dicarboxylate (**5a**–**5g**)

S. No	Compound	R	M.pt (°C)	Yield (%)
1	**5a**	–F	154	98
2	**5b**	–Cl	144	96
3	**5c**	–Me	152	94
4	**5d**	–H	164	96
5	**5e**	–Br	130	95
6	**5f**	–OMe	134	92
7	**5g**	–NO_2_	136	94

### Characterisation of compounds and their conformational studies

**Fig. 2:**
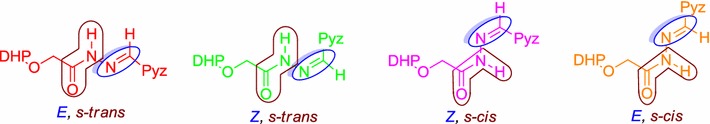
Four possible isomeric form for **5a**

The structure of these hybrids was ascertained by IR, ^1^H NMR, ^13^C NMR, and mass spectral data. The absorption signals corresponding to C=O stretching of amides appeared at 1685–1650 cm^−1^ and the NH stretching appeared in the region 3315–3244 cm^−1^ in IR spectra. It is assumed that the compound **5**, restricted rotation about imine (C=N) linkage as well as the partial double bond character of hydrazide bond led to the formation of four isomers *E*, *s*-*cis*; *E*, *s*-*trans*; *Z*, *s*-*cis* and *Z*, *s*-*trans* (Fig. 2), where *E/Z* geometrical isomers with respect to C=N double bond and *s*-*cis*/s-*trans* rotamers with respect to N–C(O) acyl hydrazide [10, 24, 25].

Literature survey also reveals that the *N*-acyl hydrazones synthesised from aromatic carbaldehyde are essentially planar and exist completely in the form of geometric (*E*)-configuration about the C=N bond due to steric hindrance on the imine bond [10, 24–27]. The NMR (^1^H and ^13^C) spectra of these hydrazones (**5a**–**5g**) also gave two sets of resonance signals which confirmed the existence of two conformational isomers in CDCl_3_ (*E*, *s*-*cis* and *E*, *s*-*trans*) and in agreement with literature, predominant isomer was assigned to the E, *s*-*cis* [10, 28–31]. Therefore, we discarded the formation of *Z*, *s*-*cis* and *Z*, *s*-*trans* isomers.

In ^1^H-NMR of acyl hydrazones (**5a**–**5g**), splitting of signals were observed for methylene (–O–C**H**_2_–), imine (N=C**H**), amide (CON**H**) and other protons which envisaged the existence of their two isomers i.e. *E*, *s*-*cis* and *E*, *s*-*trans*. For *E*, *s*-*cis* isomer, singlet for methylene (–O–C**H**_2_–) protons were observed at δ 4.54–4.61 ppm (1.65–1.70 H i.e. 82.41–85.23%). Similarly, signals for both imine (N=C**H**) proton and amide (CON**H**) proton also appeared as singlet at δ 8.32–8.74 ppm (0.83–0.85 H i.e. 83.5–85%) and δ 9.39–9.91 ppm (0.84–0.85 H i.e. 84.15–85.15%) respectively. In case of *E*, *s*-*trans* isomer singlets for methylene (–O–C**H**_2_–), imine (N=C**H**) and amide (CON**H**) protons were observed at δ 4.77–4.91 ppm (0.29–0.35 H i.e. 14.7–17.59%), 8.55–8.66 ppm (0.15–0.16 H i.e. 14.94–16.5%), 8.81–10.04 ppm (0.15–0.16 H i.e. 14.85–15.85%) respectively. The percentage of both *E*, *s*-*cis* and *E*, *s*-*trans* isomers at 25 °C were found in the range of 82–86 and 12–18%, respectively (Additional file 1: Table S1) as derived by integration area in NMR spectrum for methylene (–O–C**H**_2_–), imine (N=C**H**) and amide (CON**H**) protons.

**Fig. 3:**
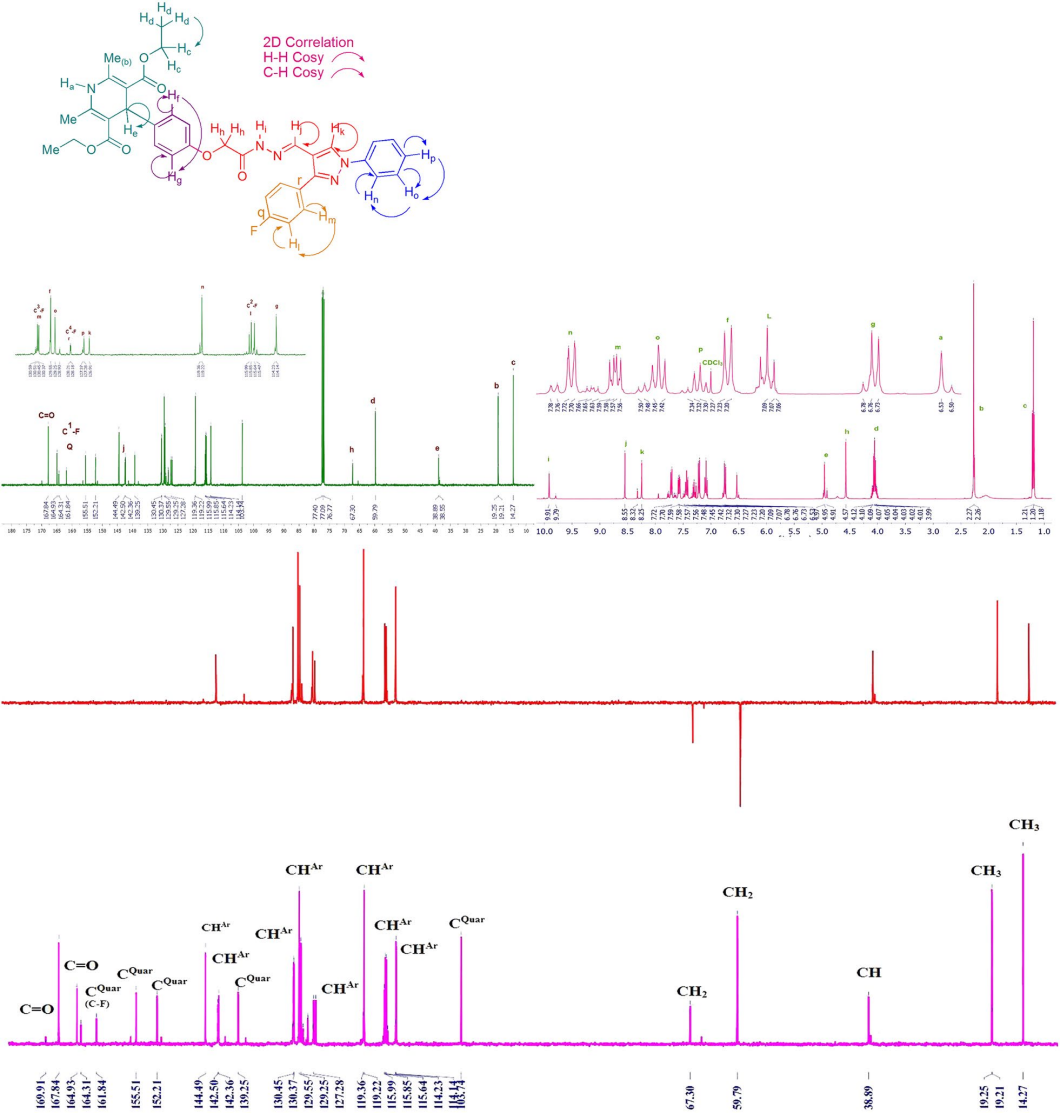
Assignment of various characteristic peaks and 2D correlation of **5a**

**Fig. 4:**
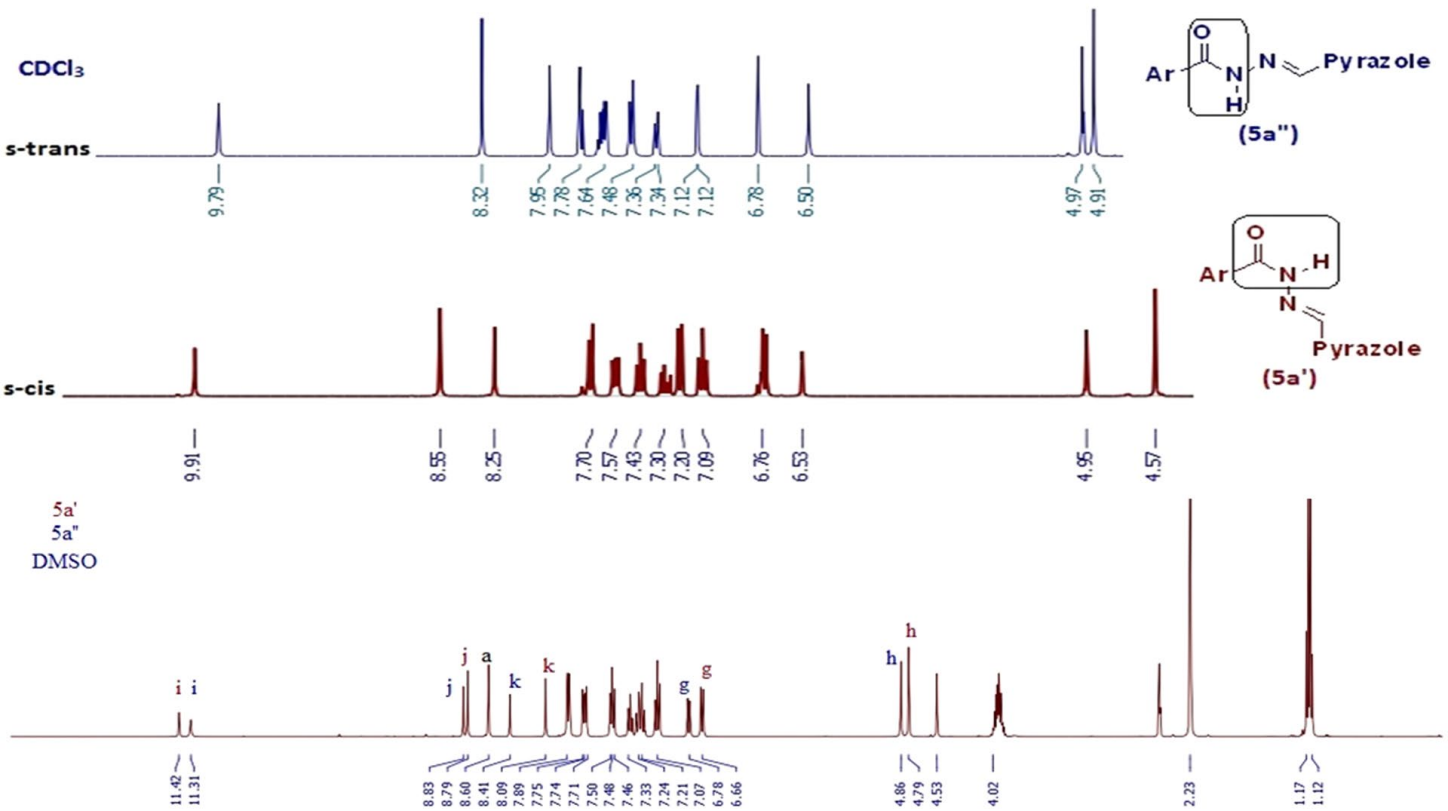
Comparison of δ of two isomers of **5a** in CDCl_3_ and DMSO

Compound **5a** was use as model to study the conformational isomers of hydrazone by means of IR, ^1^H-NMR, ^13^C-NMR, mass, ^1^H-^1^H COSY, ^1^H-^13^C HMBC spectra. In the ^1^H-NMR (Fig. 3), the protons of –OCH_2_ of test compound **5a** resonated at δ 4.57 with 85.23% abundance for *E*, *s*-*cis* conformation and at δ 4.91 with 14.77% abundance for *E*, *s*-*trans* conformation (Fig. 3) and approximately same ratio is found in the case of N=C**H** proton at δ 8.32 ppm (16.17%, *E*, *s*-*trans* conformation) and 8.55 ppm (83.83%, *E*, *s*-*cis* conformation) and for the CON**H** proton signals at δ 9.79 ppm (15.85%, *E*, *s*-*trans* conformation) and δ 9.91 ppm (84.15%, *E*, *s*-*cis* conformation). The difference between the intensities of the two signals indicates the predominant formation of *E*, *s*-*cis* isomer. In ^13^C spectra (Fig. 3), some carbons also showed two peaks instead of one, such as two peaks for –O**C**H_2_ were observed at δ 67.30 and 65.50 ppm (Fig. 3). In ESI–MS mass spectra of compound **5a**, *m/z* value was observed at 666.12 [M+H]^+^. In order to understand the effect of solvent on isomer distribution, the NMR of compound **5a** was taken in DMSO-*d*_*6*_. Interestingly the ratio for *E*, *s*-*trans* and *E*, *s*-*cis* isomers were found to be in 2:3 ratio (Fig. 4). This may be due to the solvation and stability of different conformation in different solvent.

The PM7 calculations using MOPAC2016 [32] on DELL LATITUDE E5410 on the stability of *E*, *s*-*cis* and *E*, *s*-*trans* conformation were made to corroborate the experimental results which demonstrated the higher stability of the *E, s*-*cis* isomer. Our aim was to compute semi-empirical derived properties that would be useful as starting points for understanding the ratio of conformational isomerism of *N*-acyl hydrazone in different solvent. Model compound **5a** was considered to study the geometric isomerism. As we had information about isomer distribution of **5a** compound in DMSO and CDCl_3_, we modelled *E*, *s*-*cis* and *E*, *s*-*trans* isomers to analyse the structures for conformational analysis of the amide (HNCO) group. As expected for **5a**, two minimum-energy conformers were found at about 0° and 180°, corresponding to the syn (*E*, *s*-*cis*) and anti(*E*, *s*-*trans*) arrangements. The difference in the heat of formation ∆H_f_, as calculated by the PM7 method, was found to be 13.74719 kcal/mol in CHCl_3_ and 3.17416 kcal/mol in DMSO, favouring the E, *s*-*cis* isomer. The results that we obtained are summarized in Additional file 1: Table S2. There was considerable energy difference between the *E*, *s*-*cis* and *E*, *s*-*trans* conformer in CHCl_3_ and DMSO (Additional file 1: Table S2). Thus we concluded that theoretical calculations, experimental results and literature proved that *E*, *s*-*cis* conformation was predominant conformation over *E*, *s*-*trans* conformation.

### In vitro antimalarial study

**Fig. 5:**
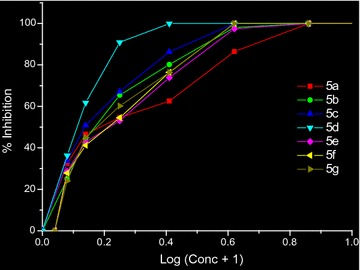
Graphical representation of % inhibition of compounds **5a**–**5g**

All the synthesised molecular hybrids of DHP and pyrazole **5a**–**5g** were screened for their in vitro anti-malarial activity against chloroquine-sensitive strain of *P. falciparum* (3D7) using chloroquine as reference drug. The number of schizonts alive at different concentrations (mg/ml) of compounds **5a**–**5g** was shown in Table 2. The results of the biological evaluation were expressed as the drug concentration resulting in 50% inhibition (IC_50_) of parasite. The antiplasmodial IC_50_ values of synthesised compounds **5a**–**5f** are depicted in Table 3. Compound **5d** was found to be most active with an IC_50_ 4.40 nM, followed by compound **5c** and **5b** with IC_50_ values of 8.08 and 8.66 nM, respectively. A comparison of % inhibition of all synthesised compounds is shown in Fig. 5. All the newly synthesised compounds were found to be twice potent except compound **5d** which is four times potent than the reference drug chloroquine.

**Table 2:** Number of alive schizont at different concentrations of compounds **5a–5g**

Drug conc. (mg/ml)	**5a**	**5b**	**5c**	**5d**	**5e**	**5f**	**5g**
0.00	113.00	108.00	110.00	110.00	106.00	105.00	105.00
0.20	91.00	81.00	84.00	70.00	82.00	81.00	85.00
0.39	75.00	60.00	65.00	42.00	67.00	67.00	63.00
0.78	66.00	46.00	47.00	10.00	56.00	53.00	47.00
1.56	57.00	30.00	26.00	0.00	34.00	30.00	30.00
3.13	30.00	2.00	0.00	0.00	9.00	0.00	3.00
6.25	0.00	0.00	0.00	0.00	0.00	0.00	0.00
12.50	0.00	0.00	0.00	0.00	0.00	0.00	0.00

**Table 3:** In vitro anti-malarial activity of diethyl 4-(4-(((3-aryl-1-phenyl-1*H*-pyrazol-4-yl)methyleneaminocarbamoyl)methoxy)phenyl)-1,4-dihydro-2,6-dimethylpyridine-3,5-dicarboxylate (**5a**–**5g**)

Compound	IC_50_ nM	IC90 nM	IC95 nM	IC99 nM
**5a**	16.87	7.98	8.64	9.14
**5b**	8.66	3.81	5.60	7.88
**5c**	8.08	3.98	4.36	4.64
**5d**	4.40	1.22	1.8	2.31
**5e**	10.49	4.72	6.21	7.87
**5f**	9.94	3.98	4.29	4.53
**5g**	9.94	3.85	5.37	7.58
**Chloroquine**	18.7	–	–	–
**Pyrimethamine**	11	–	–	–
**Artemisinin**	6	–	–	–

### In vitro antibacterial study

**Fig. 6:**
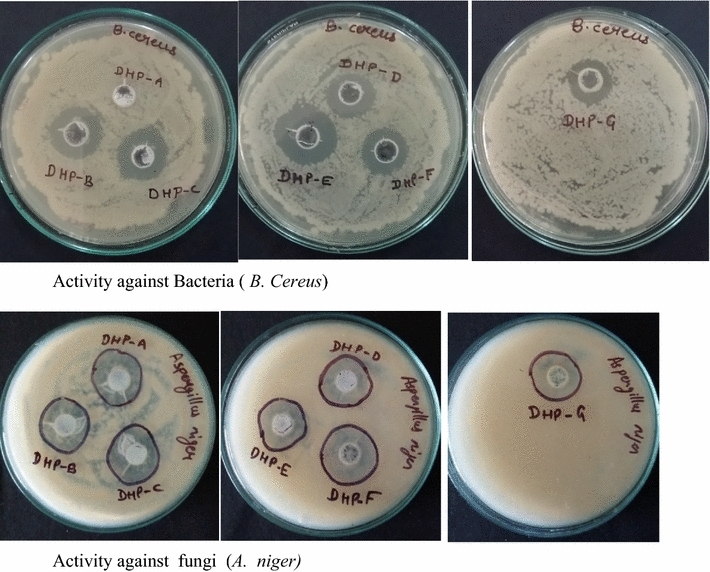
Bilogical assay for antibacterial activity. Activity against bacteria (*B. cereus*), activity against fungi (*A. niger*)

All the synthesized compounds **5a**–**5g** were evaluated in vitro for their antimicrobial activity against one Gram positive bacterium strain i.e. *Bacillus cereus*, one Gram negative bacterium strain i.e. *Escherichia coli* and antifungal activity against one yeast i.e. *Aspergillus niger* by agar well diffusion method [33]. Several compounds displayed more than 90% inhibition. As compared to reference drug Tetracycline, the acyl hydrazones **5b** (ZOI = 15 mm), **5c** (ZOI = 14 mm) and **5e** (ZOI = 17 mm) revealed very good activity against *Bacillus cereus*. As compared to reference drug clotrimazole, the compounds **5a** (ZOI = 15 mm), **5b** (ZOI = 13 mm), **5c** (ZOI = 16 mm), **5d** (ZOI = 17 mm), **5e** (ZOI = 14 mm), **5f** (ZOI = 17 mm), and **5g** (ZOI = 15 mm) revealed excellent activity against *Aspergillus niger* (Table 4; Fig. 6).

**Table 4:** Antimicrobial activity of diethyl 4-(4-(((3-aryl-1-phenyl-1*H*-pyrazol-4-yl)methyleneaminocarbamoyl)methoxy)phenyl)-1,4-dihydro-2,6-dimethylpyridine-3,5-dicarboxylate (**5a**–**5g**) by zone of inhibition method

Sample	*E. coli*	*B. cereus*	*Aspergillus niger*
	Diameter of zone of inhibition (mm)^a^		
5a	NA	11	15
5b	NA	15	13
5c	NA	14	16
5d	NA	NA	17
5e	NA	17	14
5f	NA	12	17
5g	NA	11	15
Control (tetracycline 30 mcg)	21	16	ND
Control (clotrimazole 10 mcg)	ND	ND	11

*NA* no activity

^a^Average of three samples

### In silico studies

#### Docking analysis

The complex life cycle associated with *Plasmodium falciparum* provides a number of targets which can be explored to discover new drugs for treatment of malaria. During life span, a parasite plays an important role in metabolite synthesis, membrane transport and haemoglobin degradation. Targets which are involved in these processes can be used to inhibit parasitic growth by their inhibition. Evidences indicate that the falcipain family proteases, namely FP2 and FP3 are promising targets involved in haemoglobin hydrolysis. Thus, inhibiting these targets could prevent haemoglobin hydrolysis which indeed hindered parasitic growth [34–39]. Falcipain inhibitors can be broadly divided into three categories [40]; (i) peptide based, (ii) peptidomimetic inhibitors, and (iii) non-peptidic inhibitors. Most of the falcipain inhibitors identified so far are peptide and peptidomimetic based inhibitors [40], however their utility as therapeutic agents is limited for their susceptibility due to metabolic degradation and their poor absorption through cell membranes. Thus, it would be of great interest to discover non-peptide inhibitors, which are less exposed to degradation by host proteases and thereby, more likely to offer in vivo activity. This strategy yielded several non-peptide inhibitors [41–43].

**Fig. 7:**
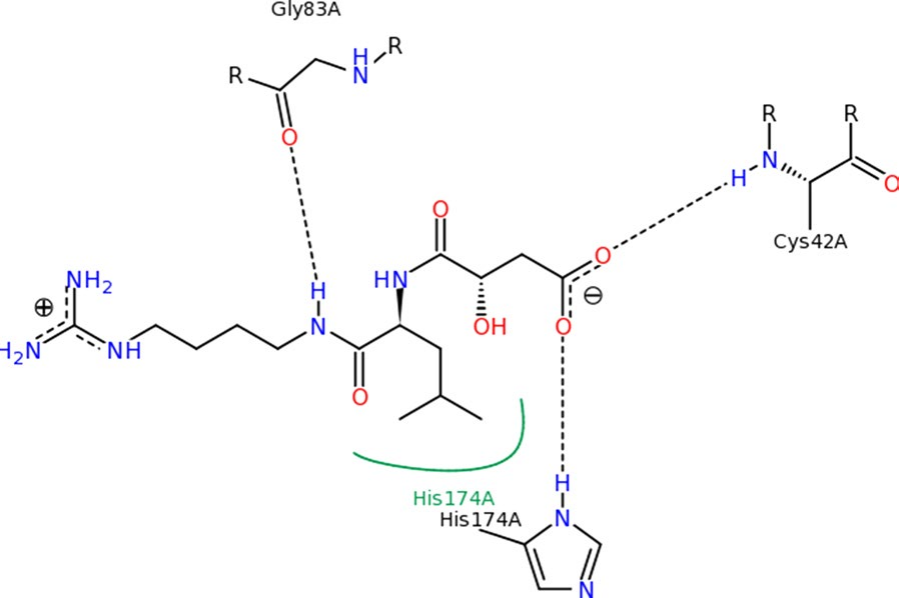
The interaction of ligand **E64** into the binding sites of **FP-2**(**PDB ID: 3BPF**)

**Fig. 8:**
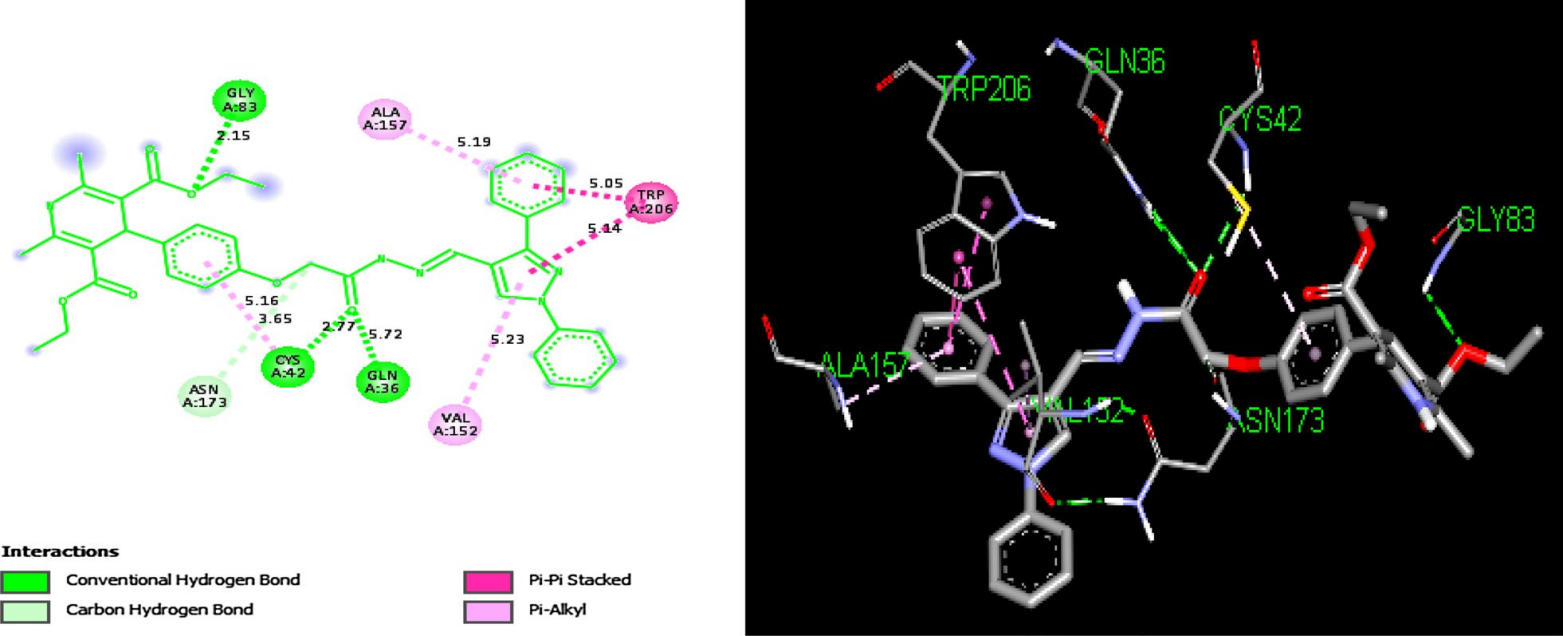
Interactions of the **5d** into the binding sites of **FP-2**(**PDB ID: 3BPF**)

In silico studies of many pyrazole based hydrazone derivatives have been revealed to inhibit malarial cysteine protease [13]. In an effort to investigate the plausible mode of action for antimalarial activity and to predict orientation of the molecules at the active site, docking simulations were performed using Auto Dock Vina program [44]. The plasmodial cysteine protease falcipain-2 is chosen for docking because it is an important target for antimalarial chemotherapy [45]. For the survival of *P. falciparum* parasite, the free amino acids are produced by hydrolysis of hemoglobin, which is carried out by trophozoites of *P. falciparum* in an acidic food vacuole [45]. The inhibition of falcipain-2 (FP2) direct to a noticeable cutback in hemoglobin hydrolysis by trophozoites. The crystal structure of falcipain-2 was co-crystallized with inhibitor E64 (*N*-[*N*-(l-3-*trans*-carboxyirane-2-carbonyl)-l-leucyl]-agmatine), which was covalently-bonded to the enzyme as to block substrates from reaching the catalytic triad as defined by Gly^83^, Cys^42^ and His^174^ residues (Fig. 7). The binding energy obtained for E64 is 7.1 kcal/mol. Docking results suggested that compound **5d** could bind the active site of falcipain-2 (Fig. 8) with binding energy of 8.5 kcal/mol. Binding energy of E64 was obtained by its re-docking non-covalently due to the limitation of available docking software in performing covalent docking. Owing to these inherent differences in the binding mechanism, thus it cannot be assumed that the DHP-pyrazole-hydrazones could possess higher potency than E64 by judging from their binding energies.

Compound **5d**, which had the highest antimalarial activity, could bind the active site of falcipain-2 via the interaction scheme shown in Fig. 8. The oxygen of ester group of dihydropyridine and acyl hydrazone form conventional hydrogen bonding with Glycine (Gly^83^) and Glutamine (Gln^36^) and Cysteine (Cys^42^) respectively. In addition, it elicited the hydrophobic interactions with other amino acids viz., Trp^206^ (pi–pi interaction), Val^152^, Ala^157^ and Cys^42^ (pi-alkyl). Methylene group made carbon hydrogen bonds with Asparagine (Asn^173^). The hydrogen bonding interaction site i.e., Gly^83^ and Cys^42^ for ligand E64 and **5d** are common (Fig. 7). The docked pose of **5d** with highest binding affinity is shown in Fig. 8. It can be noticed from Fig. 8 that mainly hydrogen bonding and hydrophobic interactions (pi–pi interaction and pi-alkyl interaction) are responsible for fixing of the compound **5d**. Some important interactions of **5d** with different amino acids have been listed in Additional file 1: Table S3. The role of Cys^42^ residue in the inhibition of FP2 by E64 has been well known in literature [46, 47]. All these facts show that binding of compound **5d** to these active site residues might be the cause of antimalarial activity. The docking results suggested that the antimalarial activity of the DHP-pyrazole-hydrazone derivatives might be due to their inhibitions of falcipain-2.

## Conclusion

1,4-dihydropyridin-4-yl-phenoxyacetohydrazides with differently substituted pyrazole carbaldehyde were synthesised using molecular hybridisation. The resulting compound (**5**) exists in two conformations (i.e., *E*, *s*-*cis* and *E*, *s*-*trans*) as revealed from conformational studies. All the synthesised compounds were screened for their in vitro antimalarial activities against chloroquine-sensitive malaria parasite *P. falciparum 3D7* and exhibit good inhibition as compared to standard drug chloroquine. In vitro antiplasmodial IC_50_ value of compound **5d** was found to be 4.40 nM which is lower than that of all the three reference drugs chloroquine (18.7 nM), Pyrimethamine (11 nM) and Artimisinin (6 nM). In silico binding study of compound **5d** with plasmodial cysteine protease falcipain-2 shows that inhibition of falcipain-2 could be the probable reason for the potency shown by compound **5d**. The results obtained from in vitro and in silico studies suggest that these compounds can be used as potent anti-malarial agents after their cytotoxicity evaluation. All the synthesized compounds **5a**–**5g** show moderate to good antimicrobial activity against Gram negative bacterium strain i.e. *Escherichia coli* and excellent antifungal activity against *Aspergillus niger* compared to reference drug.

## Experimental

All the chemicals used were purchased from Spectrochem, Avra and Sigma Aldrich and were used as received. Silica gel 60 F_254_ (Precoated aluminium plates) from Merck was used to monitor reaction progress. Melting points were determined on Buchi Melting Point M-560 apparatus and are uncorrected. IR (KBr) spectra were recorded on Perkin Elmer FTIR spectrophotometer and the values are expressed as ν_max_ cm^−1^. The ^1^H and ^13^C spectra were recorded on Bruker top spin and Jeol JNM ECX-400P at 400 MHz and 100 MHz respectively. Mass spectra were recorded at Bruker Micro TOF Q-II. The chemical shift values are recorded on δ scale and the coupling constants (J) are in Hertz. Pyrazole carbaldehydes were prepared according to the procedure described in literature [48].

### General procedure for synthesis of acyl hydrazones (**5a**–**5g**)

To a clear solution of **3** (1.00 mmol) in ethanol (10 ml), 1.00 mmol of pyrazole carbaldehyde (**4**) and a catalytic amount of glacial acetic acid were added and the reaction mixture was refluxed for 10 h. The progress of the reaction was monitored by TLC using ethyl acetate-petroleum ether, (70:30, v/v). After completion, the reaction mixture was poured onto crushed ice. The precipitate formed was collected by vacuum filtration and washed with cold ethanol to afford pure products (**5a**–**5g**) in 92–98% yield. The products were characterized by IR, ^1^H NMR, ^13^C NMR and Mass spectra.

#### *Diethyl4*-*(4*-*(((3*-*(4*-*fluorophenyl)*-*1*-*phenyl*-*1H*-*pyrazol*-*4*-*yl)methyleneaminocarbamoyl) methoxy)phenyl)*-*1,4*-*dihydro*-*2,6*-*dimethylpyridine*-*3,5*-*dicarboxylate* (**5a**)

White solid; M.p.: 154 °C; Yield 98%; IR (KBr, cm^−1^) ν_max_: 3488, 3244, 3087, 1669, 1483, 1225, 1010; ^1^H NMR (400 MHz, CDCl_3_) δ 9.91 (s, 1H, CONH, 84.15%), 9.79 (s, 1H, CONH, 15.85%), 8.55 (s, 1H, NH=CH, 83.83%), 8.32 (s, 1H, NH=CH, 16.17%), 8.25 (s, 1H, Pyr–H, 84.69%), 7.95 (s, 1H, Pyr–H, 15.31%), 7.80–7.75 (m, 2H, ArH(n), 16.66%), 7.74–7.69 (m, 2H, ArH(n), 83.34%), 7.67–7.61 (m, 2H, ArH(m), 16.44%), 7.61–7.53 (m, 2H, ArH(m), 83.56%), 7.51–7.41 (m, 2H, ArH(o)), 7.32 (m, 1H, ArH(p)), 7.21 (m, 2H, ArH(f)), 7.10 (m, 2H, ArH(L)), 6.77 (m, 2H, ArH(g), 18.13%), 6.75 (m, 2H, ArH(g), 81.87%), 6.53 (s, 1H, NH, 83.16%), 6.50 (s, 1H, NH, 16.84%), 4.97 (s, 1H, C_4_–H, 15.74%), 4.95 (s, 1H, C_4_–H, 84.26%), 4.91 (s, 2H, –OCH_2_, 14.77%), 4.57 (s, 2H, –OCH_2_, 85.23%), 4.12–3.99 (m, 4H, CH_2_), 2.27 (s, 6H, CH_3_, 83.20%), 2.26 (s, 6H, CH_3_, 16.80%), 1.20 (t, J = 7.1 Hz, 6H, CH_3_); ^13^C NMR (100 MHz, CDCl_3_) δ 167.84, 164.93, 164.31, 161.84, 156.40, 155.51, 152.21, 144.49, 142.50, 142.36, 139.25, 130.59, 130.51, 130.45, 130.37, 129.55, 129.25, 128.93, 128.21, 128.18, 127.37, 127.28, 126.91, 119.36, 119.22, 116.16, 115.99, 115.85, 115.64, 115.47, 114.23, 114.14, 103.74, 77.40, 77.09, 76.77, 67.30, 59.79, 38.89, 38.55, 19.25, 19.21, 14.27. MS (*m/z*) 666.12; Anal. Calcd. For C_37_H_36_FN_5_O_6_: C, 66.76; H, 5.45; F, 2.85; N, 10.52; O, 14.42. Found: C, 66.81; H, 5.39; F, 2.91; N, 10.47; O, 14.37.

#### *Diethyl4*-*(4*-*(((3*-*(4*-*chlorophenyl)*-*1*-*phenyl*-*1H*-*pyrazol*-*4*-*yl)methyleneaminocarbamoyl) methoxy)phenyl)*-*1,4*-*dihydro*-*2,6*-*dimethylpyridine*-*3,5*-*dicarboxylate* (**5b**)

White solid; M.p.: 144 °C; Yield 96%; IR (KBr, cm^−1^) ν_max_: 3488, 3244, 2987, 1655, 1483, 1255; ^1^H NMR (400 MHz, CDCl_3_) δ 9.42 (s, 1H, CONH), 8.74 (s, 1H, NH=CH, 14.71%), 8.66 (s, 1H, NH=CH, 85.29%), 8.33 (s, 1H, Pyr–H, 16.4%), 8.16 (s, 1H, Pyr–H, 83.6%), 7.77 (m, 2H, ArH), 7.59 (m, 2H, ArH), 7.51–7.47 (m, 2H, ArH), 7.46 (m, 2H, ArH), 7.34 (m, 1H, ArH), 7.23 (m, 2H, ArH), 6.79 (m, 2H, ArH), 5.66 (s, 1H, NH, 83.33%), 5.64 (s, 1H, NH, 16.67%), 4.95 (s, 1H, C_4_–H, 16.82%), 4.93 (s, 1H, C_4_–H, 83.18%), 4.61 (s, 2H, –OCH_2_, 84.5%), 4.54 (s, 2H, –OCH_2_, 15.5%), 4.11–4.03 (m, 4H, CH_2_), 2.31 (s, 6H, CH_3_), 1.21 (t, *J* = 7.1 Hz, 6H, CH_3_); ^13^C NMR (100 MHz, CDCl_3_) δ 167.77, 164.74, 155.44, 144.09, 142.44, 142.14, 139.33, 134.91, 130.01, 129.71, 129.47, 129.13, 127.49, 127.23, 119.37, 116.03, 114.17, 104.10, 77.45, 77.13, 76.81, 67.33, 59.91, 38.97, 19.65, 14.38; Anal. Calcd. for C_37_H_36_ClN_5_O_6_: C, 65.14; H, 5.32; Cl, 5.20; N, 10.27; O, 14.07. Found: C, 65.09; H, 5.27; Cl, 5.16; N, 10.31; O, 14.11.

#### *Diethyl4*-*(4*-*(((3*-*(4*-*methylphenyl)*-*1*-*phenyl*-*1H*-*pyrazol*-*4*-*yl)methyleneaminocarbamoyl) methoxy)phenyl)*-*1,4*-*dihydro*-*2,6*-*dimethylpyridine*-*3,5*-*dicarboxylate* (**5c**)

White solid; M.p.: 152 °C; Yield 94%; IR (KBr, cm^−1^) ν_max_: 3317, 1669, 1512, 1211, 1096, 752; ^1^H NMR (400 MHz, CDCl_3_) δ 9.37 (s, 1H, CONH), 8.69 (s, 1H, NH=CH, 15.46%), 8.65 (s, 1H, NH=CH, 84.54%), 8.33 (s, 1H, Pyr–H, 15%), 8.15 (s, 1H, Pyr–H, 85%), 7.81–7.74 (m, 2H, ArH), 7.53 (m, 2H, ArH), 7.46 (m, 2H, ArH), 7.33 (m, 1H, ArH), 7.29 (m, 2H, ArH), 7.23 (m, 2H, ArH), 6.79 (m, 2H, ArH), 5.71 (s, 1H, NH, 84.11%), 5.66 (s, 1H, NH, 15.89%), 4.97 (s, 1H, C_4_–H, 17.27%), 4.93 (s, 1H, C_4_–H, 82.73%), 4.77 (s, 2H, –OCH_2_, 17.59%), 4.60 (s, 2H, –OCH_2_, 82.41%), 4.12–4.01 (m, 4H, CH_2_), 2.41 (s, 3H, CH_3_, 84.90%), 2.39 (s, 3H, CH_3_, 15.10%), 2.31 (s, 6H, CH_3_, 84.85%), 2.30 (s, 6H, CH_3_, 15.15%), 1.20 (t, *J* = 7.1 Hz, 6H, CH_3_); ^13^C NMR (100 MHz, CDCl_3_) δ 167.77, 164.64, 155.52, 152.83, 144.19, 142.72, 142.43, 139.73, 139.02, 129.60, 129.45, 128.67, 127.25, 126.89, 119.34, 115.88, 114.17, 103.98, 77.45, 77.14, 76.82, 59.86, 38.97, 21.42, 19.56, 14.38; Anal. Calcd. For C_38_H_39_N_5_O_6_: C, 68.97; H, 5.94; N, 10.58; O, 14.51. Found: C, 69.01; H, 5.89; N, 10.62; O, 14.47.

#### *Diethyl 4*-*(4*-*(((1,3*-*diphenyl*-*1H*-*pyrazol*-*4*-*yl)methyleneaminocarbamoyl)methoxy)phenyl)*-*1,4*-*dihydro*-*2,6*-*dimethylpyridine*-*3,5*-*dicarboxylate* (**5d**)

White solid; M.p.: 164 °C; Yield 96%; IR (KBr, cm^−1^) ν_max_: 3276, 2987, 1672, 1499, 1211, 1095, 750; ^1^H NMR (400 MHZ, CDCl_3_) δ 10.04 (s, 1H, CONH, 15%), 9.39 (s, 1H, CONH, 85%), 8.68 (s, 1H, NH=CH, 13.4%), 8.55 (s, 1H, NH=CH, 86.6%), 8.35 (s, 1H, Pyr–H, 15%), 8.16 (s, 1H, Pyr–H, 85%), 7.79 (m, 2H, ArH), 7.64 (m, 2H, ArH), 7.50 (m, 2H, ArH), 7.47 (m, 1H, ArH), 7.46–7.41 (m, 2H, ArH), 7.33 (m, 1H, ArH), 7.23 (m, 2H, ArH), 6.79 (m, 2H, ArH), 5.65 (s, 1H, NH, 85%), 5.63 (s, 1H, NH, 15%), 4.97 (s, 1H, C_4_–H, 17.39%), 4.93 (s, 1H, C_4_–H, 82.61%), 4.61 (s, 2H, –OCH_2_), 4.11–4.02 (m, 4H, CH_2_), 2.31 (s, 6H, CH_3_, 83.69%), 2.30 (s, 6H, CH_3_, 16.31%), 1.20 (t, *J* = 7.1 Hz, 6H, CH_3_); ^13^C NMR (100 MHz, CDCl_3_) δ 167.67, 143.87, 142.42, 130.33, 129.68, 129.51, 128.96, 128.82, 119.38, 114.18, 104.24, 92.62, 77.44, 77.12, 76.80, 67.53, 67.34, 61.71, 59.88, 39.15, 19.74, 14.38; Anal. Calcd. for C_37_H_37_N_5_O_6_: C, 68.61; H, 5.76; N, 10.81; O, 14.82. Found: C, 68.57; H, 5.81; N, 10.93; O, 14.74.

#### Diethyl 4-(4-(((3-(4-bromophenyl)-1-phenyl-1H-pyrazol-4-yl)methyleneaminocarbamoyl) *methoxy)phenyl)*-*1,4*-*dihydro*-*2,6*-*dimethylpyridine*-*3,5*-*dicarboxylate* (**5e**)

White solid; M.p.: 130 °C; Yield 95%; IR (KBr, cm^−1^) ν_max_: 3517, 3317, 3087, 1684, 1483, 1211, 1096, 767; ^1^H NMR (400 MHz, CDCl_3_) δ 9.44 (s, 1H, CONH), 8.81 (s, 1H, NH=CH, 16.33%), 8.65 (s, 1H, NH=CH, 83.67%), 8.33 (s, 1H, Pyr–H, 16.5%), 8.16 (s, 1H, Pyr–H, 83.5%), 7.77 (m, 2H, ArH), 7.63–7.59 (m, 2H, ArH), 7.52 (m, 2H, ArH), 7.50–7.45 (m, 2H, ArH), 7.34 (m, 1H, ArH), 7.24 (m, 2H, ArH), 6.79 (m, 2H, ArH), 5.69 (s, 1H, NH, 82.35%), 5.67 (s, 1H, NH, 17.65%), 4.95 (s, 1H, C_4_–H, 17.65%), 4.93 (s, 1H, C_4_–H, 82.35%), 4.61 (s, 2H, –OCH_2_, 84.65%), 4.54 (s, 2H, –OCH_2_, 15.35%), 4.11–4.03 (m, 4H, CH_2_), 2.31 (s, 6H, CH_3_, 80.61%), 2.30 (s, 6H, CH_3_, 19.39%), 1.21 (t, *J* = 7.1 Hz, 6H, CH_3_); ^13^C NMR (100 MHz, CDCl_3_) δ 184.71, 167.74, 164.71, 155.45, 151.38, 144.06, 142.44, 142.12, 139.33, 132.15, 132.07, 131.98, 131.89, 131.09, 130.53, 130.28, 129.84, 129.70, 129.46, 128.24, 127.49, 127.21, 119.84, 119.36, 116.04, 114.19, 104.12, 77.44, 77.12, 76.81, 67.35, 59.89, 38.99, 19.64, 14.39; Anal. Calcd. For C_37_H_36_BrN_5_O_6_: C, 61.16; H, 4.99; Br, 11.00; N, 9.64; O, 13.21. Found: C, 61.13; H, 5.01; Br, 10.96; N, 9.67; O, 13.17.

#### *Diethyl4*-*(4*-*(((3*-*(4*-*methoxyphenyl)*-*1*-*phenyl*-*1H*-*pyrazol*-*4*-*yl)methyleneaminocarbamoyl) methoxy)phenyl)*-*1,4*-*dihydro*-*2,6*-*dimethylpyridine*-*3,5*-*dicarboxylate* (**5f**)

White solid; M.p.: 134 °C; Yield 92%; IR (KBr, cm^−1^) ν_max_: 3317, 1684, 1497, 1197, 1096; ^1^H NMR (400 MHz, CDCl_3_) δ 9.44 (s, 1H, CONH, 84.16%), 8.81 (s, 1H, CONH, 15.84%), 8.64 (s, 1H, NH=CH, 83.96%), 8.51 (s, 1H, NH=CH, 16.04%), 8.32 (s, 1H, Pyr–H, 15.15%), 8.15 (s, 1H, Pyr–H, 84.85%), 7.78–7.75 (m, 2H, ArH), 7.59–7.53 (m, 2H, ArH), 7.45 (m, 2H, ArH), 7.31 (m, 1H, ArH), 7.21 (m, 2H, ArH), 7.02–6.98 (m, 2H, ArH), 6.80–6.74 (m, 2H, ArH), 5.80 (s, 1H, NH), 4.97 (s, 1H, C_4_–H, 16.95%), 4.93 (s, 1H, C_4_–H, 83.05%), 4.60 (s, 2H, –OCH_2_, 85.4%), 4.58 (s, 2H, –OCH_2_, 14.6%), 4.11-4.01 (m, 4H, CH_2_), 3.87 (s, 3H, –OCH_3_, 17.67%), 3.85 (s, 3H, –OCH_3_, 82.33%), 2.30 (s, 6H, CH_3_, 82.21%), 2.29 (s, 6H, CH_3_, 17.79%), 1.20 (t, *J* = 7.1 Hz, 6H, CH_3_); ^13^C NMR (101 MHz, CDCl_3_) δ 167.73, 164.60, 160.18, 155.46, 153.25, 144.09, 142.73, 142.43, 139.47, 130.35, 130.04, 129.76, 129.64, 129.46, 127.22, 126.90, 124.58, 119.81, 119.30, 115.76, 114.35, 114.28, 114.19, 104.10, 77.45, 77.13, 76.81, 67.36, 59.87, 55.47, 38.97, 19.60, 14.38; Anal. Calcd. For C_38_H_39_N_5_O_7_: C, 67.34; H, 5.80; N, 10.33; O, 16.52. Found: C, 67.31; H, 5.85; N, 10.29; O, 16.48.

#### *Diethyl4*-*(4*-*(((3*-*(4*-*nitrophenyl)*-*1*-*phenyl*-*1H*-*pyrazol*-*4*-*yl)methyleneaminocarbamoyl) methoxy)phenyl)*-*1,4*-*dihydro*-*2,6*-*dimethylpyridine*-*3,5*-*dicarboxylate* (**5g**)

Yellow solid; M.p.: 136 °C; Yield 94%; IR (KBr, cm^−1^) ν_max_: 3317, 1655, 1497, 1325, 1211, 1082, 853, 752; ^1^H NMR (400 MHz, CDCl_3_) δ 9.59 (s, 1H, CONH, 85.15%), 9.21 (s, 1H, CONH, 14.85%), 8.66 (s, 1H, NH=CH, 85.11%), 8.36 (s, 1H, NH=CH, 14.89%), 8.33 (s, 1H, Pyr–H, 14.94%), 8.32 (s, 1H, Pyr–H, 85.06%), 8.30 (m, 2H, ArH), 7.90–7.84 (m, 2H, ArH), 7.78 (m, 2H, ArH), 7.49 (m, 2H, ArH), 7.37 (m, 1H, ArH), 7.22 (m, 2H, ArH), 6.78 (m, 2H, ArH), 5.82 (s, 1H, NH, 15.53%), 5.78 (s, 1H, NH, 84.47%), 4.93 (s, 1H, C_4_–H, 84.62%), 4.92 (s, 1H, C_4_–H, 15.38%), 4.89 (s, 2H, –OCH_2_, 15.42%), 4.61 (s, 2H, –OCH_2_, 84.58%), 4.11–4.01 (m, 4H, CH_2_), 2.31 (s, 6H, CH_3_, 14.85%), 2.30 (s, 6H, CH_3_, 85.15%), 1.20 (t, *J* = 7.1 Hz, 6H, CH_3_); ^13^C NMR (101 MHz, CDCl_3_) δ 167.79, 164.96, 155.48, 147.77, 144.13, 142.44, 141.60, 139.15, 138.70, 129.78, 129.42, 129.35, 127.83, 127.74, 124.09, 119.43, 116.79, 114.20, 104.05, 77.44, 77.12, 76.81, 67.41, 59.92, 38.99, 19.62, 14.38; Anal. Calcd. For C_37_H_36_N_6_O_8_: C, 64.15; H, 5.24; N, 12.13; O, 18.48. Found: C, 64.19; H, 5.28; N, 12.17; O, 18.44.

### Anti-malarial activity

#### Parasite cultivation

The anti-malarial activity of synthesised acyl hydrazones (**5a**–**5g**) was assessed against chloroquine-sensitive *P. falciparum* (3D7) isolate. *P. falciparum* was cultivated in human A Rh+ red blood cells using RPMI 1640 medium (Sigma, India) supplemented with AB Rh+ serum (10%), 5% sodium bicarbonate (Sigma, India) and 40 μg/mL of gentamycin sulphate 17 (Sigma, India).

#### In vitro test for anti-malarial activity

The in vitro activity of *P. falciparum* intra erythrocytic stage on synthesised compounds was evaluated by Schizonts maturation Inhibition (SMI) method [49]. Accordingly, the compounds were dissolved in DMSO and serially diluted with RPMI 1640 medium to reach 1 mg/ml before use. The cultures, before testing, were synchronized by treatment with 5% d-sorbitol with a parasitemia of 0.6–0.8%. Each well received 10 μl of parasite-infected erythrocytes, 5% hematocrit and 90 μl of different compound dilutions. Chloroquine and solvent controls contained similar volumes of the solvent, as that of test wells. The plates were incubated at 37 °C for 24 h. After confirmation of the presence of 10% mature schizonts in control wells, the blood from each well was harvested and a thick film was prepared on a glass slide. The blood films were stained for 40 min with Giemsa stain at a dilution of 10% in double distilled water. Three independent optical-microscopy readings of the number of schizonts with three or more nuclei were carried out in 200 parasitized red blood cells for each dilution and duplicate. Growth inhibition was expressed as the percentage of schizonts in each concentration, compared with controls.

#### Calculation and analysis

The number of schizonts counted per well was directly entered into the nonlinear regression software, HN NonLin V 1.1 [50], which was particular for the analysis of in vitro drug sensitivity assay for malaria. Individual dose response curves were generated and their IC_50_ values were determined.

### Antimicrobial activity

#### Test microorganisms

3 microbial strains were selected on the basis of their clinical importance in causing diseases in humans. One Gram-positive bacteria (*Bacillus cereus*); one Gram-negative bacteria (*Escherichia coli*) and one yeast, (*Aspergillus niger*) were screened for evaluation of antibacterial and antifungal activities of the synthesized pyrazoles. All the microbial cultures were procured from Microbial Type Culture Collection (MTCC), IMTECH, Chandigarh. The bacteria were sub cultured on nutrient agar whereas yeast on malt yeast agar.

### In-vitro antibacterial activity

The antimicrobial activity of synthesised acyl hydrazones (**5a**–**5g**) was evaluated by the agar-well diffusion method. All the microbial cultures were adjusted to 0.5 McFarland standard, which is visually comparable to a microbial suspension of approximately 1.5 × 10^8^ cfu/ml. Agar medium (20 ml) was poured into each Petri plate and plates were swabbed with 100 µl inocula of the test microorganisms and kept for 15 min for adsorption. Using sterile cork borer of 8 mm diameter, wells were created into the seeded agar plates which were loaded with a 100 µl volume with concentration of 8.0 mg/ml of each compound reconstituted in the dimethylsulphoxide (DMSO). All the plates were incubated at 37 °C for 24 h. Antimicrobial activity of each compound was evaluated by measuring the zone of growth inhibition against the test organisms with zone reader (Hi Antibiotic zone scale). DMSO was used as a negative control whereas Tetracycline was used as positive control for bacteria and clotrimazole for fungi. This procedure was performed in triplicates for each organism.

### Computational details

#### Energy calculation with PM7 Hamiltonian method

All calculation was carried out with PM7 Hamiltonian method using MOPAC2016 program [32] on DELL LATITUDE E5410. All chemical structures were drawn in Marvin Sketch 15.12.7.0 [51]. The structures under study were optimized using default value of GNORM and properties were calculated. The calculations were performed in solution phase using chloroform (dielectric constant = 4.8) and dimethylsulfoxide (dielectric constant = 46.70) solvents in Andreas Klamt’s COSMO implicit solvation model.

#### Docking studies

The crystal structure of plasmodial cysteine protease falcipain-2 was obtained from the Brookhaven Protein Data Bank http://www.rcsb.org/pdb (PDB entry: 3BPF). To carry outdocking studies, the 2D-structures of **5d** were drawn in Marvin Sketch 15.12.7.0 [51]. Then explicit hydrogens were added and this was converted to 3D and its energy was minimized. Co-crystallized ligand was removed from pdb file 3BPF and protein molecule was prepared by deleting solvent molecules using UCSF Chimera 1.10 [52]. Incomplete side chains were replaced using Dun Brack Rotamer library [53]. Hydrogens were added and gasteiger charges were calculated using AMBERff14SB and antechamber [54]. The prepared file was saved as pdb format and used for further studies. These structures of ligand **5d** and proteins were transformed into pdbqt format with Auto Dock Tools [55]. Docking studies were carried out by using Auto Dock Vina 1.1.2 [44]. Grid center was placed on the active site. The centers and sizes of grid box were as follows: center_x = − 58.5196350008, center_y = − 1.19310953271 and center_z = − 17.0068559885, size_x = 25.0, and size_y = 25.0, size_z = 25.0. Exhaustiveness of the global search algorithm was set to be 100. Then, finally docking results were viewed in Discovery Studio Visualizer 16.1.0.15350 [56].

## Additional file

**Additional file 1.** Additional tables.

## References

[1] World Malaria Report 2014 (2014) World Health Organization

[2] Alílio MS, Bygbjerg IC, Breman JG (2004). Are multilateral malarial research and control programs the most successful? Lessons from the past 100 years in Africa. Am J Trop Med Hyg.

[3] Towie N (2006). Malaria breakthrough raises spectre of drug resistance. Nature.

[4] Tumwebaze P, Conrad MD, Walakira A, LeClair N, Byaruhanga O, Nakazibwe C, Kozak B, Bloome J, Okiring J, Kakuru A, Bigira V, Kapisi J, Legac J, Gut J, Cooper RA, Kamya MR, Havlir DV, Dorsey G, Greenhouse B, Nsobya SL, Rosenthal PJ (2015). Impact of antimalarial treatment and chemoprevention on the drug sensitivity of malaria parasites isolated from Ugandan children. Antimicrob Agents Chemother.

[5] Fancony C, Brito M, Gil JP (2016). *Plasmodium falciparum* drug resistance in Angola. Malar J.

[6] Sirawaraporn W, Sathitkul T, Sirawaraporn R, Yuthavong Y, Santi DV (1997). Antifolate-resistant mutants of *Plasmodium falciparum* dihydrofolate reductase. Proc Natl Acad Sci USA.

[7] Jnr CV, Danuello A, Bolzani VS, Barreiro EJ, Fraga CAM (2007). Molecular hybridization: a useful tool in the design of new drug prototypes. Curr Med Chem.

[8] Walsh JJ, Bell A (2009). Hybrid drugs for malaria. Curr Pharm Des.

[9] Ullooraa S, Shabarayab R, Ranganathanc R, Adhikari AV (2013). Synthesis, anticonvulsant and anti-inflammatory studies of new 1,4-dihydropyridin-4-yl phenoxy acetohydrazones. Eur J Med Chem.

[10] Syakaev V, Podyachev S, Buzykin B, Latypov S, Habicher W, Konovalov A (2006). NMR study of conformation and isomerization of aryl- and hetero arylaldehyde 4-tert-butylphenoxyacetylhydrazones. J Mol Struct.

[11] Cunico W, Cechinel CA, Bonacorso HG, Martins AP (2006). Antimalarial activity of 4-(5-trifluoromethyl-1H-pyrazol-1-yl)-chloroquine analogues. Bioorg Med Chem Lett.

[12] Bekhit AA, Hymete A, Asfaw H, Bekhit AD (2012). Synthesis and biological evaluation of some pyrazole derivatives as anti-malarial agents. Arch Pharm.

[13] Santanna CMR, Alencastro RB, Rodrigues CR, Barreiro G, Barreiro EJ, Neto JDM, Freitas ACC (1996). A semi empirical study of pyrazole acylhydrazones as potential antimalarial agents. Int J Quantum Chem.

[14] Sugimoto N, Watanabe H, Ide A (1960). The synthesis of l-α-amino-β-(pyrazolyl-N)-propionic acid in *Citrullus vulgaris*. Tetrahedron.

[15] Hannah J, Kelly K, Patchett AA, Steelman SL, Morgan ER (1975). Substituted pyrazolo corticoids as topical antiinflam-matory agents. J Med Chem.

[16] Stauffer SR, Huang YR, Aron ZD, Coletta CJ, Sun J, Katzenellenbogen BS, Katzenellenbogen JA (2001). Triarylpyrazoles with basic side chains: development of pyrazole-based estrogen receptor antagonists. Bioorg Med Chem.

[17] Fink BE, Mortensen DS, Stauffer SR, Aron ZD, Katzenellenbogen JA (1999). 1,3,5-triaryl-4-alkyl-pyrazoles bind to the estrogen receptor (ER) with high affinity. Chem Biol.

[18] Stauffer SR, Coletta CJ, Tedesco R, Nishiguchi G, Carlson K, Sun J, Katzenellenbogen BS, Katzenellenbogen JA (2000). Pyrazole ligands: structure-affinity/activity relationships and estrogen receptor-alpha-selective agonists. J Med Chem.

[19] Ashton WT, Hutchins SM, Greenlee WJ, Doss GA, Chang RS, Lotti VJ, Faust KA, Chen TB, Zingaro GJ (1993). Nonpeptide angiotensin II antagonists derived from 1H-pyrazole-5-carboxylates and 4-aryl-1H-imidazole-5-carboxylates. J Med Chem.

[20] Abdel-Aziz M, Abuo-Rahma GEDA, Hassan AA (2009). Synthesis of novel pyrazole derivatives and evaluation of their antidepressant and anticonvulsant activities. Eur J Med Chem.

[21] Manikannan R, Venkatesan R, Muthusubramanian S, Yogeeswari P, Sriram D (2010). Pyrazole derivatives from azines of substituted phenacyl aryl/cyclohexylsulfides and their antimycobacterial activity. Bioorg Med Chem Lett.

[22] Verissimo E, Berry N, Gibbons P, Cristiano ML, Rosenthal PJ, Gut J, Ward SA, Neill PM (2008). Design and synthesis of novel 2-pyridone peptidomimetic falcipain 2/3 inhibitors. Bioorg Med Chem Lett.

[23] Desai PV, Patny A, Sabnis Y, Tekwani B, Gut J, Rosenthal PJ, Srivastava A, Avery M (2004). Identification of novel parasitic cysteine protease inhibitors using virtual screening. The ChemBridge database. J Med Chem.

[24] Patorski P, Wyrzykiewicz E, Bartkowiak G (2013). Synthesis and conformational assignment of N-(E)-stilbenyloxymethylenecarbonyl-substituted hydrazones of acetone and *o*-(m- and p-) Chloro-(nitro-)benzaldehydes by means of and NMR spectroscopy. J Spectroscopy.

[25] Ershov AY, Lagoda IV, Yakimovich SI, Pakalnis VV, Zerova IV, Dobrodumov AV, Shamanin VV (2009). Tautomerism and conformational isomerism of mercaptoacetylhydrazones of aliphatic and aromatic aldehydes. Russ J Org Chem.

[26] Gonzaga DTG, Silva FCD, Ferreira VF, Wardell JL, Wardell SMSV (2016). Crystal structures of 1-Aryl-1H- and 2-Aryl-2H-1,2,3-triazolyl hydrazones, conformational consequences of different classical hydrogen bonds. J Braz Chem Soc.

[27] Glidewell C, Low JN, Skakle JMS, Wardell JL (2004). Hydrogen bonding in nitroaniline analogues: 4-nitrobenzaldehyde hydrazone forms hydrogen-bonded sheets of R 4 4(26) rings. Acta Crystallogr Sect C: Cryst Struct Commun.

[28] Hamzi I, Barhoumi-Slimi TM, Abidi R (2016). Synthesis, characterization, and conformational study of acylhydrazones of α, β-unsaturated aldehydes. Heteroat Chem.

[29] Angelusiu MV, Barbuceanu SF, Draghici C, Almajan GL (2010). New Cu(II), Co(II), Ni(II) complexes with aroyl-hydrazone based ligand. Synthesis, spectroscopic characterization and in vitro antibacterial evaluation. Eur J Med Chem.

[30] Stadler AM, Harrowfield J (2009). Bis-acyl-/aroyl-hydrazones as multidentate ligands. Inorg Chim Acta.

[31] Onnis V, Cocco MT, Fadda R, Congiu C (2009). Synthesis and evaluation of anticancer activity of 2-arylamino-6-trifluoromethyl-3-(hydrazonocarbonyl)pyridines. Bioorg Med Chem.

[32] Stewart JP (2016) Stewart computational chemistry, MOPAC2016, Version: 16.043 W web: http://OpenMOPAC.net

[33] Aneja KR, Sharma C, Joshi R (2011). In vitro efficacy of amaltas (*Cassia fistula* L.) against the pathogens causing otitis externa. J Microbiol.

[34] Drinkwater N, Vinh NB, Mistry SN, Bamert RS, Ruggeri C, Holleran JP, Loganathan S, Paiardini A, Charman SA, Powell AK, Avery AK, McGowan S, Scammells PJ (2016). Potent dual inhibitors of *Plasmodium falciparum* M1 and M17 aminopeptidases through optimization of S1 pocket interactions. Eur J Med Chem.

[35] Singh A, Rosenthal PJ (2001). Comparison of efficacies of cysteine protease inhibitors against five strains of *Plasmodium falciparum*. Antimicrob Agents Chemother.

[36] Sharma M, Chauhan K, Srivastava RK, Singh SV, Srivastava K, Saxena JK, Puri SK, Chauhan PMS (2014). Design and synthesis of a new class of 4-aminoquinolinyl- and 9-anilinoacridinyl schiff base hydrazones as potent antimalarial agents. Chem Biol Drug Des.

[37] Sharma RK, Younis Y, Mugumbate G, Njoroge M, Gut J, Rosenthal PJ, Chibale K (2015). Synthesis and structure-activity-relationship studies of thiazolidinediones as antiplasmodial inhibitors of the *Plasmodium falciparum* cysteine protease falcipain-2. Eur J Med Chem.

[38] Rosenthal PJ, Olson JE, Lee GK, Palmer JT, Klaus JL, Rasnick D (1996). Antimalarial effects of vinyl sulfone cysteine proteinase inhibitors. Antimicrob Agents Chemother.

[39] Shenai BR, Lee BJ, Alvarez-Hernandez A, Chong PY, Emal CD, Neitz RJ, Roush WR, Rosenthal PJ (2003). Structure-activity relationships for inhibition of cysteine protease activity and development of *Plasmodium falciparum* by peptidyl vinyl sulfones. Antimicrob Agents Chemother.

[40] Ettari R, Bova F, Zappala M, Grasso S, Micale N (2010). Falcipain-2 inhibitors. Med Res Rev.

[41] Sajid M, McKerrow JH (2002). Cysteine proteases of parasitic organisms. Mol Biochem Parasitol.

[42] Schirmeister T, Kaeppler U (2003). Non-peptidic inhibitors of cysteine proteases. Mini Rev Med Chem.

[43] Melnyk P, Leroux V, Sergheraert C, Grellier P (2006). Design, synthesis and in vitro antimalarial activity of an acylhydrazone library. Bioorg Med Chem Lett.

[44] Trott O, Olson AJ, Vina AD (2010). Improving the speed and accuracy of docking with a new scoring function, efficient optimization and multithreading. J Comput Chem.

[45] Kerr ID, Lee JH, Pandey KC, Harrison A, Sajid M, Rosenthal PJ, Brinen LS (2009). Structures of falcipain-2 and falcipain-3 bound to small molecule inhibitors: implications for substrate specificity. J Med Chem.

[46] Grazioso G, Legnani L, Toma L, Ettari R, Micale N, Micheli CD (2012). Mechanism of falcipain-2 inhibition by a, b-unsaturated benzo[1,4]diazepin-2-one methyl ester. J Comput Aided Mol Des.

[47] Arafet K, Ferrer S, Martí S, Moliner V (2014). Quantum mechanics/molecular mechanics studies of the mechanism of falcipain-2 inhibition by the epoxysuccinate E64. Biochemistry.

[48] Kumar P, Kumar S, Husain K, Kumar A (2011). An efficient synthesis of pyrazolechalcones under solvent free conditions at room temperature. Chin Chem Lett.

[49] Lambros C, Vanderberg JP (1979). Synchronization of *Plasmodium falciparum* erythrocytic stages in culture. J Parasitol.

[50] Noedl H (2002) Non linear evaluation of malaria drug sensitivity data (HN-NonLin V1.1) Bangkok, Thailand: Armed Forces Research Institute for Medical Sciences. http://www.meduniwien.ac.at/user/harald.noedl/malaria/download.html

[51] Mavin Sketch 15.12.7.0 ChemAxon Ltd (1998–2015) http://www.chemaxon.com

[52] Pettersen Goddard TD, Huang CC, Couch GS, Greenblatt DM, Meng EC, Ferrin TE (2004). UCSF Chimera—a visualization system for exploratory research and analysis. J Comput Chem.

[53] Dunbrack RL (2002). Rotamer library in the 21st century. Curr Opin Struct Boil.

[54] Wang J, Wang W, Kollman PA, Case DA (2006). Automatic atom type and bond type perception in molecular mechanical calculations. J Mol Graphics Modeling.

[55] Forte S, AutoDock Tools (version 1.5.6 rc2) (1999–2010) Molecular Graphics Laboratory, Department of Molecular Biology, The Scripps Research Institute, 1999–2010. http://mgltools.scripps.edu

[56] Discovery Studio 2016 client (2005–2016) Accelrys Software Inc.

