# Correction to: Design, synthesis, conformational and molecular docking study of some novel acyl hydrazone based molecular hybrids as antimalarial and antimicrobial agents

**DOI:** 10.1186/s13065-018-0374-9

**Published:** 2018-02-06

**Authors:** Parvin Kumar, Kulbir Kadyan, Meenakshi Duhan, Jayant Sindhu, Vineeta Singh, Baljeet Singh Saharan

**Affiliations:** 1 Department of Chemistry, Kurukshetra University, Kurukshetra, 136119 India; 2 S D (PG) College, Panipat, 132103 India; 3 National Institute of Malaria Research, Dwarka, New Delhi 110077 India; 4 Department of Microbiology, Kurukshetra University, Kurukshetra, 136119 India

## Correction to: Chemistry Central Journal (2017) 11:115 10.1186/s13065-017-0344-7

After publication of the original article [1], the following error was reported in the Results section of the Abstract: “antifungal activity against one yeast i.e. *Aspergillus niger*” should read: “antifungal activity against one fungus i.e. *Aspergillus niger*”. The authors would like to confirm all antifungal activity has been screened against fungi not yeast.

This error has been corrected in the original article [1].
