# Supplementary material for: Design, synthesis, conformational and molecular docking study of some novel acyl hydrazone based molecular hybrids as antimalarial and antimicrobial agents
Source: Chem Cent J. 2017 Nov 14;11:115. doi: 10.1186/s13065-017-0344-7 (PMC5686033; doi:10.1186/s13065-017-0344-7)
Supplement: Supplementary file 1 — Additional file 1. Additional tables. [file 13065_2017_344_MOESM1_ESM.docx]

**Design, synthesis, conformational and molecular docking study of some novel acyl hydrazone based molecular hybrids as antimalarial and antimicrobial agents**

**Parvin Kumar^1*^, Kulbir Kadyan^1^, Meenakshi Duhan^1^, Jayant Sindhu^2^, Vineeta Singh^3^, Baljeet Singh Saharan^4^**

^1^Department of Chemistry, Kurukshetra University Kurukshetra-136119

^2^S D (PG) College, Panipat-132103

^3^National Institute of Malaria Research, Dwarka, New Delhi-110077

^4^Department of Microbiology, Kurukshetra University Kurukshetra-136119

[*E.mail: parvinjangra@gmail.com](mailto:*E.mail:%20parvinjangra@gmail.com); parvinchem@kuk.ac.in

**Procedure for synthesis of diethyl 4-(4-hydroxyphenyl)-2,6-dimethyl-1,4-dihydropyridine-3,5-dicarboxylate (1) [1]** A mixture of 4-hydroxybenzaldehyde (1.00 mmol) ethyl acetoacetate (2.00 mmol) and ammonium acetate (2.00 mmol) was refluxed in ethanol for 6-8 hrs. Progress of reaction was monitored by TLC using petroleum ether: Ethyl acetate (60:40, *v/v*). The reaction mixture was poured into crushed ice and solid then separated was filtered, washed with water and dried under vacuum. The product was recrystallized using ethanol. Yield 95%, M.p. 239-241 ^o^C.

**Procedure for synthesis of diethyl 4-(4-(2-ethoxy-2-oxoethoxy)phenyl)-2,6-dimethyl-1,4-dihydropyridine-3,5- dicarboxylate (2) [1]** To a clear solution of **1** (2.00 mmol) in Acetone: DMF (2:3, *v/v*), ethyl bromoacetate (2.00 mmol) and K_2_CO_3_ (2.50 mmol) were added with stirring. The reaction mixture was stirred at 80°C for 8 hours. The progress of the reaction was monitored by TLC using petroleum ether: Ethyl acetate (60:40, *v/v*). After completion of reaction as monitored by TLC, reaction mixture was poured in a beaker containing crushed ice. The solid so obtained was filtered using suction, washed with water and dried. The crude product so obtained was recrystallized using hot ethanol to afford pure product **2** in 94% yield. M.p.105-107^o^C; IR (ʋ_max_, cm^-1^): 3351, 2979, 2905, 1739, 1686, 1221; ^1^H NMR (DMSO-*d_6_*, 400 MHz, δ ppm): 8.73 (s, 1H), 7.03 (d, 2H, *J*=6.8 Hz), 6.73 (d, 2H, *J*=6.8 Hz), 4.78 (s, 1H), 4.66 (s, 2H), 4.13 (m, 2H), 3.96 (m, 4H), 2.23 (s, 6H), 1.18 (t, 3H, *J*=7.2 Hz), 1.12 (t, 3H, *J*=6.8 Hz).

**Procedure for synthesis of 2-(4-(3,5-bis(ethoxycarbonyl)-2,6-dimethyl-1,4-dihydropyridin-4-yl)phenoxy)acetic acid hydrazide (3) [1]** To a clear solution of **2** (1.00 mmol) in ethanol (25 mL), hydrazine hydrate (20 mmol) was added and the mixture was refluxed for 4 hrs. The progress of the reaction was monitored by TLC using petroleum ether: Ethyl acetate (30:70, *v/v*). The reaction mixture was quenched using ice and the solid so obtained was filtered using suction, dried and recrystallized using ethanol to afford pure product **3** in 98% yield. M.p. 191-193^o^C

**Table S1**: Isomeric % of compounds **5a-5g** on the basis of ^1^H NMR.

| Compound | **CONH** | **NH=CH** | **Pyr-H** | **DHPNH** | **C_4_-H** | **-OCH_2_** | **-CH_3_** |
| --- | --- | --- | --- | --- | --- | --- | --- |
| **5a** |  | | | | | | |
| *s-cis* | 9.91 (84.15%) | 8.55 (83.83%) | 8.25 (84.69%) | 6.53 (83.16%) | 4.95 (84.26%) | 4.57 (85.23%) | 2.27 (83.20%) |
| *s-trans* | 9.79 (15.85%) | 8.32 (16.17%) | 7.95 (15.31%) | 6.50 (16.84%) | 4.97 (15.74%) | 4.91(14.77%) | 2.26 (16.80%) |
| **5b** |  | | | | | | |
| *s-cis* | 9.42^a^ | 8.66 (85.29%) | 8.16 (83.6%) | 5.66 (83.33%) | 4.93 (83.18%) | 4.61 (84.5%) | 2.27 (83.20%) |
| *s-trans* |  | 8.74 (14.71%) | 8.33 (16.4%) | 5.64 (16.67%) | 4.95 (16.82%) | 4.54 (15.5%) | 2.26 (16.80%) |
| **5c** |  |  |  |  |  |  |  |
| *s-cis* | 9.40^a^ | 8.65 (84.54%) | 8.15 (85%) | 5.71 (84.11%) | 4.93 (82.73%) | 4.60 (82.41%) | 2.31 (84.85%) |
| *s-trans* |  | 8.69 (15.46%) | 8.33 (15%) | 5.66 (15.89%) | 4.97 (17.27%) | 4.77 (17.59%) | 2.30 (15.15%) |
| **5d** |  |  |  |  |  |  |  |
| *s-cis* | 9.39 (85%) | 8.55 (86.6%) | 8.16 (85%) | 5.65 (85%) | 4.93 (82.61%) | 4.61^a^ | 2.31 (83.69%) |
| *s-trans* | 10.04 (15%) | 8.68 (13.4%) | 8.35 (15%) | 5.63 (15%) | 4.97 (17.39%) |  | 2.30 (16.31%) |
| **5e** |  | | | | | | |
| *s-cis* | 9.44^a^ | 8.65 (83.67%) | 8.16 (83.5%) | 5.69 (82.35%) | 4.93 (82.35%) | 4.61 (84.65%) | 2.31 (80.61%) |
| *s-trans* |  | 8.81 (16.33%) | 8.33 (16.5%) | 5.67 (17.65%) | 4.95 (17.65%) | 4.54 (15.35%) | 2.30 (19.39%) |
| **5f** |  |  |  |  |  |  |  |
| *s-cis* | 9.44 (84.16%) | 8.64 (83.96%) | 8.15 (84.85%) | 5.80^a^ | 4.93 (83.05%) | 4.60 (85.4%) | 2.30 (82.21%) |
| *s-trans* | 8.81 (15.84%) | 8.51 (16.04%) | 8.32 (15.15%) |  | 4.97 (16.95%) | 4.58 (14.6%) | 2.29 (17.79%) |
| **5g** |  | | | | | | |
| *s-cis* | 9.59 (85.15%) | 8.66 (85.11%) | 8.32 (85.06%) | 5.78 (84.47%) | 4.93 (84.62%) | 4.61 (84.58%) | 2.30 (85.15%) |
| *s-trans* | 9.21 (14.85%) | 8.36 (14.89%) | 8.33 (14.94%) | 5.82 (15.53%) | 4.92 (15.38%) | 4.89 (15.42%) | 2.31 (14.85%) |

^a^singlet observed

**Table S2.** Computational studies MOPAC calculations.

| **Calculation** | ***s-trans* CHCl_3_** | ***s-trans* DMSO** | ***s-cis* DMSO** | ***s-cis* CHCl_3_** |
| --- | --- | --- | --- | --- |
| Heat of formation (kcal/mol) | -155.24361 | -165.33130 | -168.50546 | -168.99080 |
| Total energy (kcal/mol) | -189044.76013 | -189054.81148 | -189049.50201 | -189049.36414 |
| Energy of atoms (kcal/mol) | 188914.28101 | 188914.28101 | 188914.28101 | 188914.28101 |
| Sum (kcal/mol) | -130.47912 | -140.53048 | -135.22100 | -135.08313 |
| Dispersion energy (kcal/mol) | -24.73269 | -24.73214 | -33.37160 | -33.91724 |
| H-bond energy (kcal/mol) | -0.08914 | -0.07965 | 0.08715 | 0.00957 |
| Mm corr. For -CO-NH- (kcal/mol) | 0.05733 | 0.01096 | 0.0000 | 0.00000 |
| Sum kcal/mol | -155.24361 | -165.33130 | -168.50546 | -168.99080 |
| Total energy (ev) | 25007.23795 | 7849.62087 | 11211.19247 | 30185.19609 |
| Electronic energy (ev) | -1.10042 | -1.76196 | -1.66658 | -1.09172 |
| Core-core repulsion (ev) | 6.81736 | 5.97103 | 8.57234 | 1.86008 |
| Dielectric energy (ev) | 9.52113 | 10.30736 | 5.94557 | 3.34179 |
| Gadient norm | 8.780849 | 8.770257 | 8.683215 | 8.694397 |
| Dipole debye | -8.781; -1.040 | -8.770; -1.056 | -8.683; -1.027 | -8.694; -0.996 |
| Ionization potential (ev) | 1107.07 | 1073.70 | 976.58 | 1005.63 |
| Homo lumo energies (ev) | 1407.26 | 1368.93 | 1471.05 | 1484.70 |
| Cosmo area square angstroms | -155.24361 | -165.33130 | -168.50546 | -168.99080 |
| Cosmo volume cubic angstroms | -189044.76013 | -189054.81148 | -189049.50201 | -189049.36414 |

**Table S3**: Some important interactions of **5d** with different amino acids.

| **Name** | **Distance (Å)** | **Category** | **Type** |
| --- | --- | --- | --- |
| A:GLN36:HE21 - :5D:O | 2.81383 | Hydrogen Bond | Conventional Hydrogen Bond |
| A:GLN36:HE22 - :5D:O | 2.4724 | Hydrogen Bond | Conventional Hydrogen Bond |
| A:CYS42:HN - :5D:O | 2.7662 | Hydrogen Bond | Conventional Hydrogen Bond |
| A:GLY83:HN - :5D:O | 2.15329 | Hydrogen Bond | Conventional Hydrogen Bond |
| :5D:C - A:ASN173:O | 3.64554 | Hydrogen Bond | Carbon Hydrogen Bond |
| A:TRP206 - :5D | 4.22003 | Hydrophobic | Pi-Pi Stacked |
| A:TRP206 - :5D | 3.70619 | Hydrophobic | Pi-Pi Stacked |
| :5D - A:TRP206 | 5.13917 | Hydrophobic | Pi-Pi Stacked |
| :5D - A:VAL152 | 5.2294 | Hydrophobic | Pi-Alkyl |
| :5D - A:ALA157 | 5.18822 | Hydrophobic | Pi-Alkyl |
| :5D - A:CYS42 | 5.158 | Hydrophobic | Pi-Alkyl |

^1^H NMR spectrum of **5a**


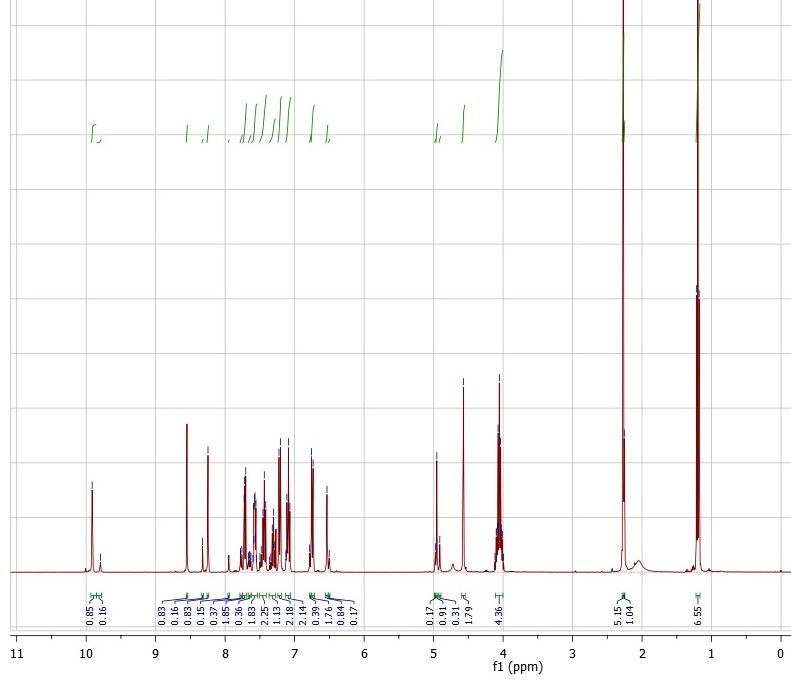


^13^C NMR spectrum of **5a**


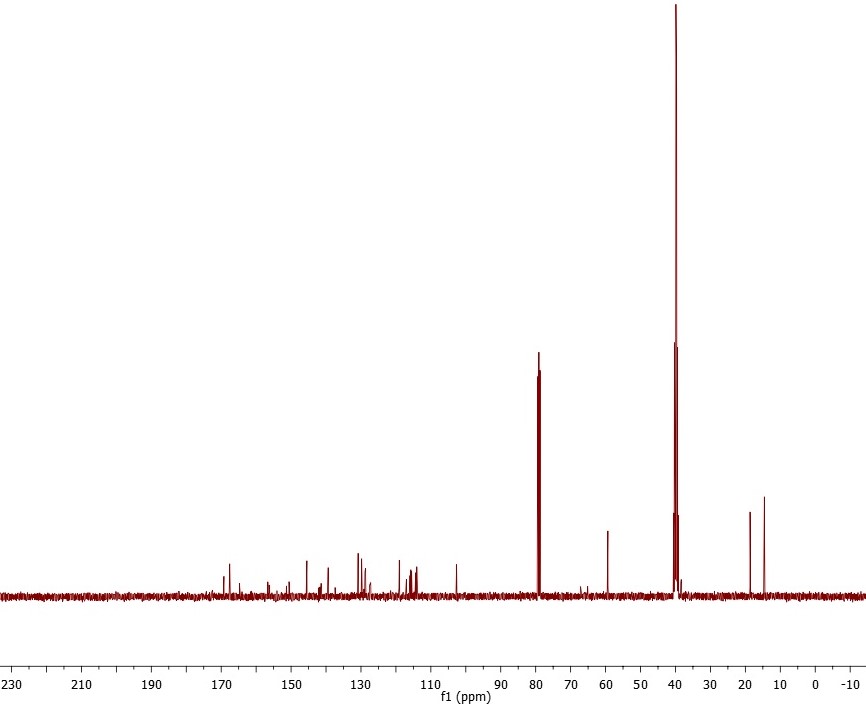


Mass spectrum of **5a**


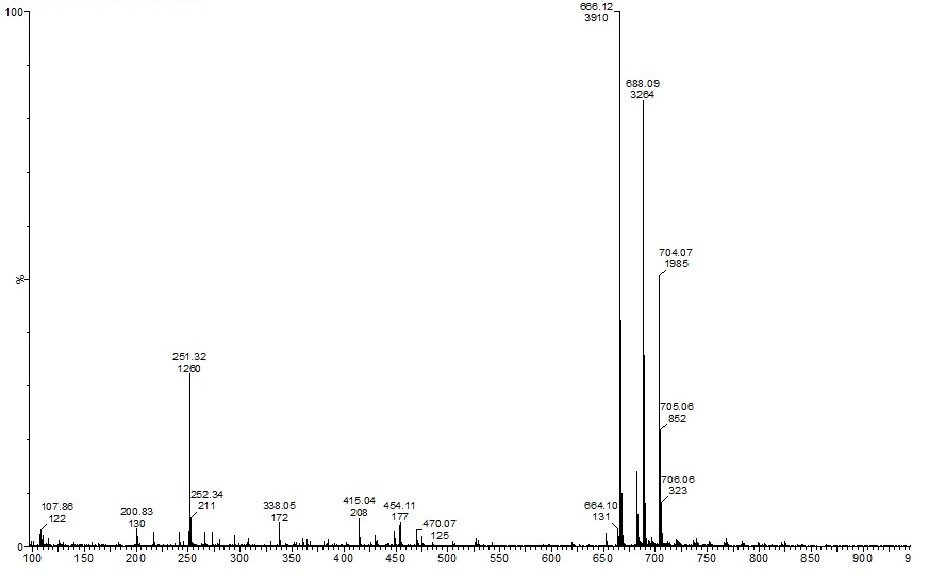


^1^H NMR spectrum of **5b**
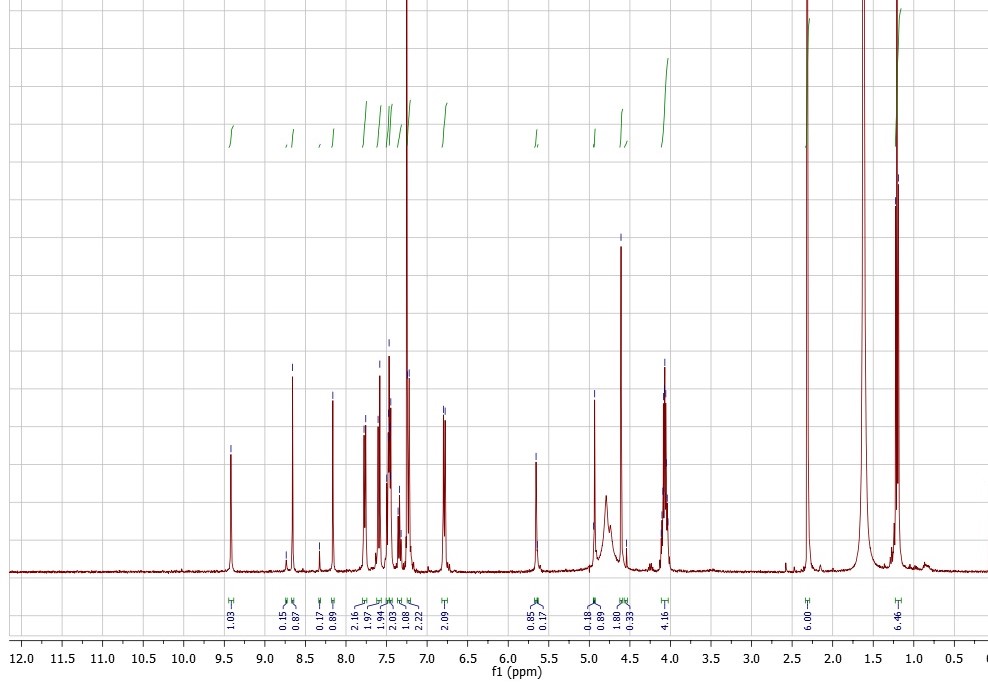


^13^C NMR spectrum of **5b**


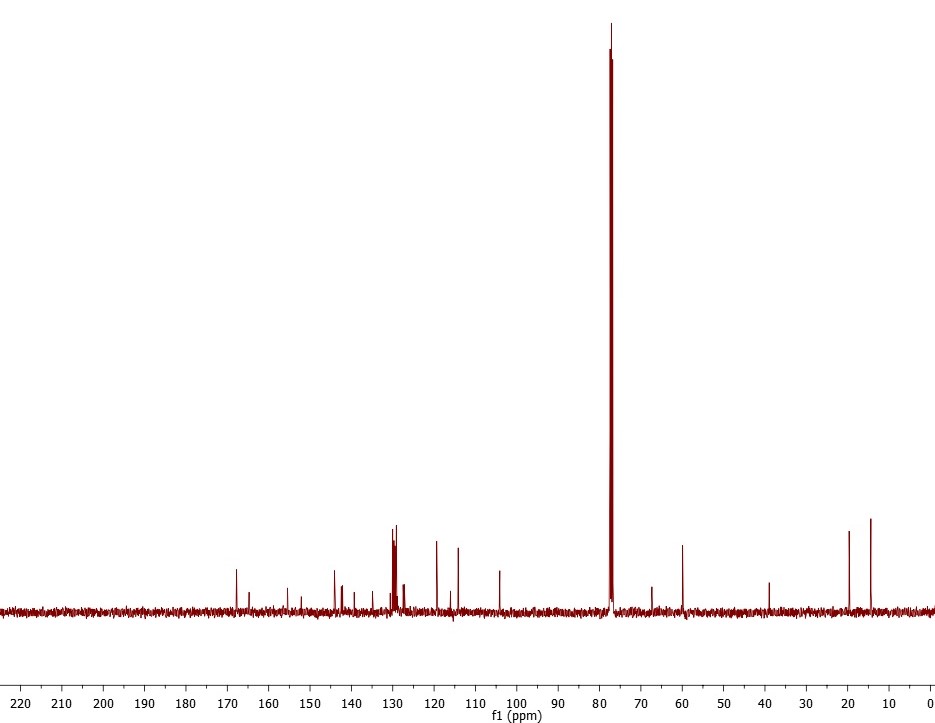


^1^H NMR spectrum of **5c**


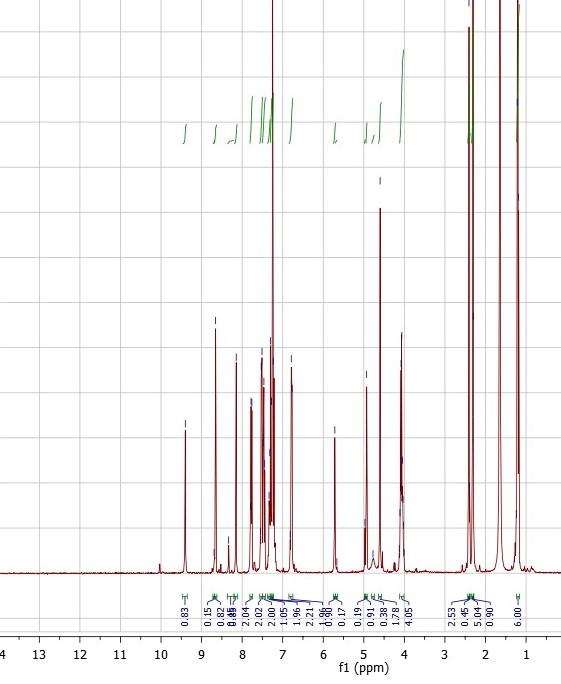


^13^C NMR spectrum of **5c**


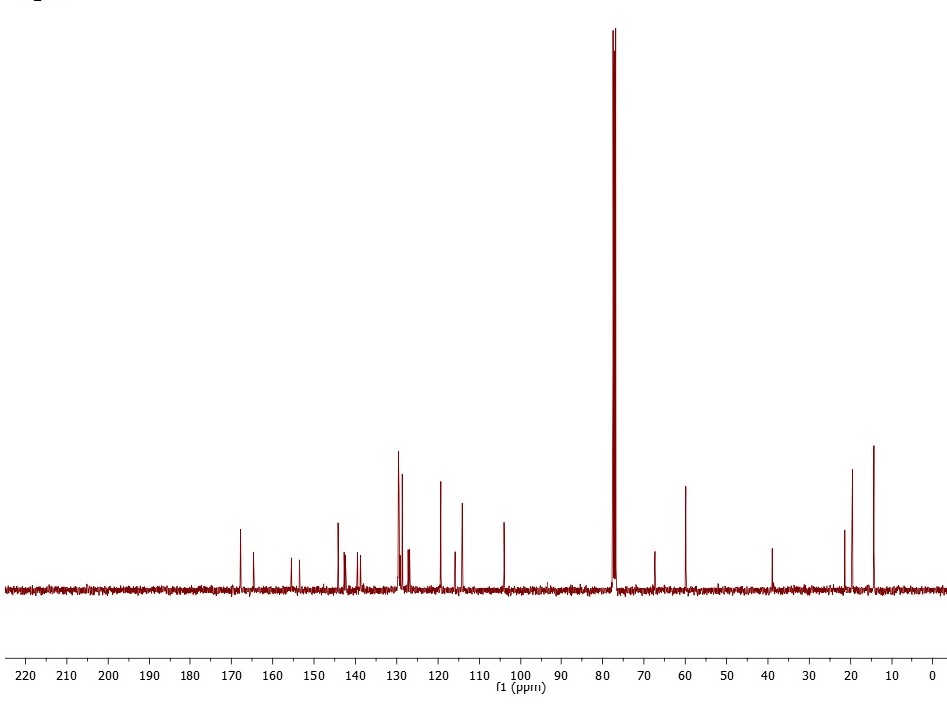


^1^H NMR spectrum of **5d**
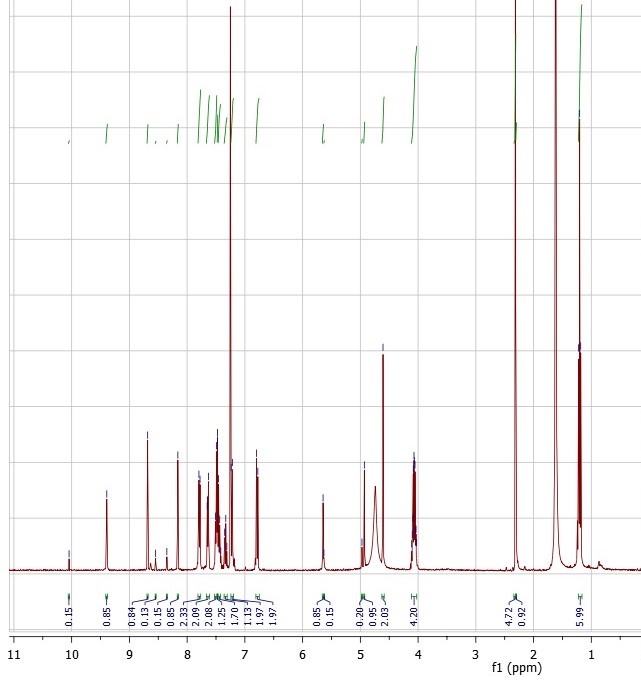


^13^C NMR spectrum of **5d**


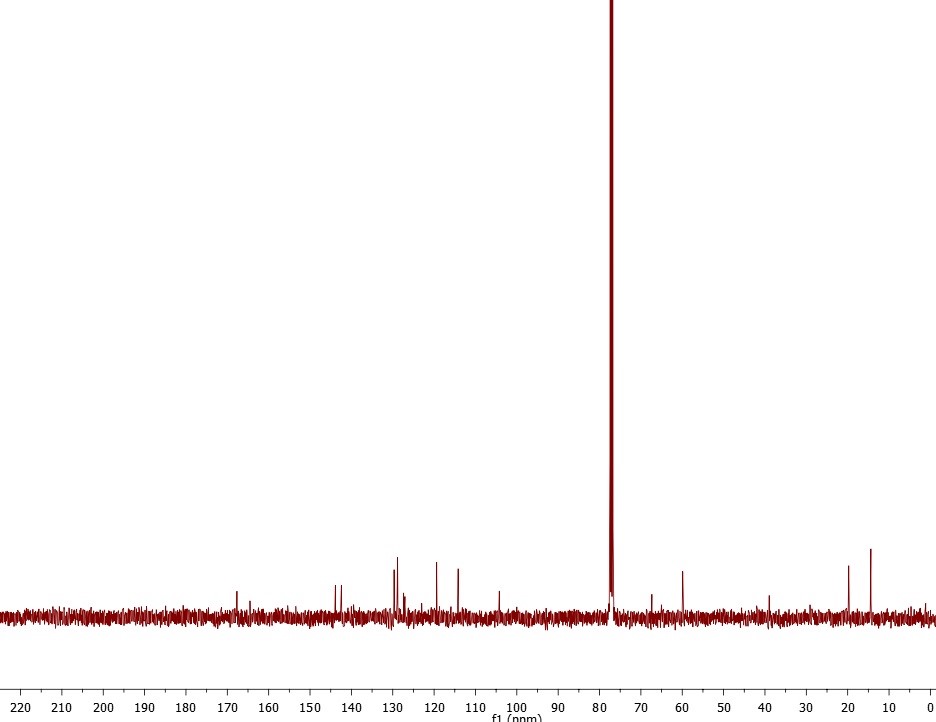


^1^H NMR spectrum of **5e**


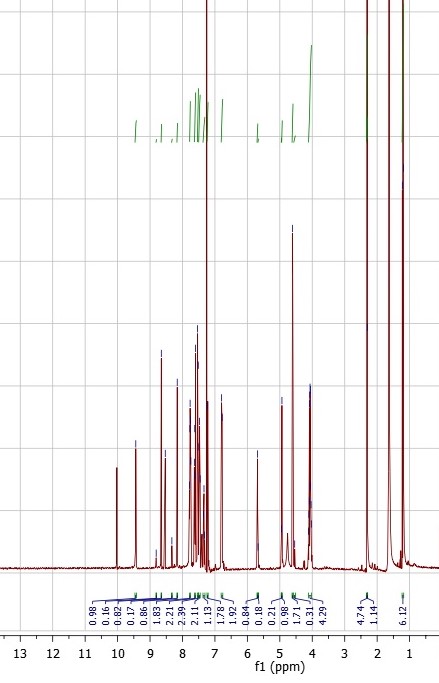


^3^C NMR spectrum of **5e**


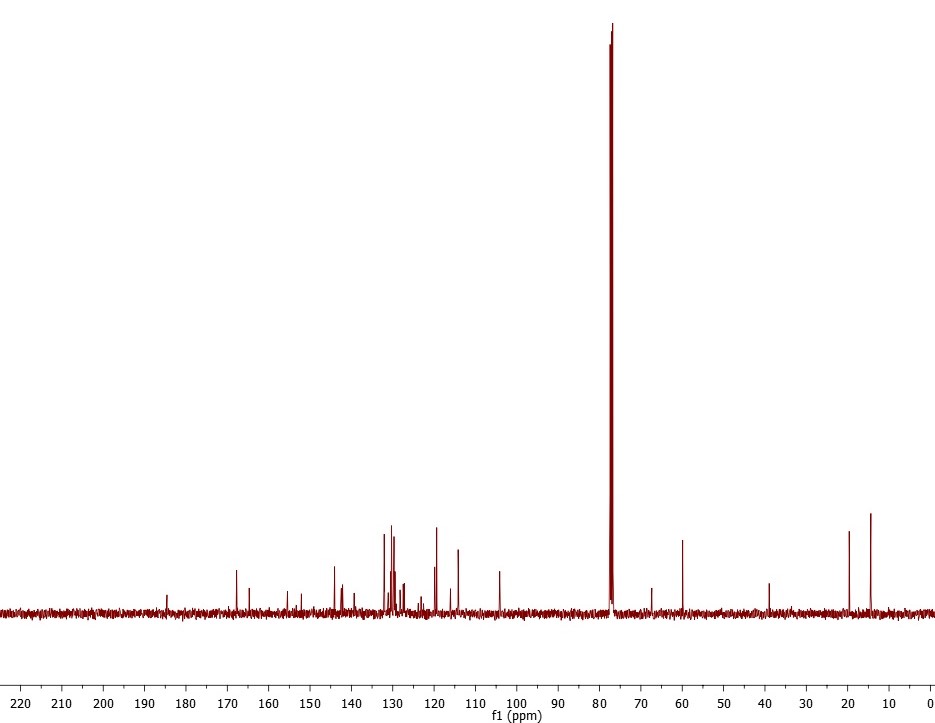


^1^H NMR spectrum of **5f**^1^


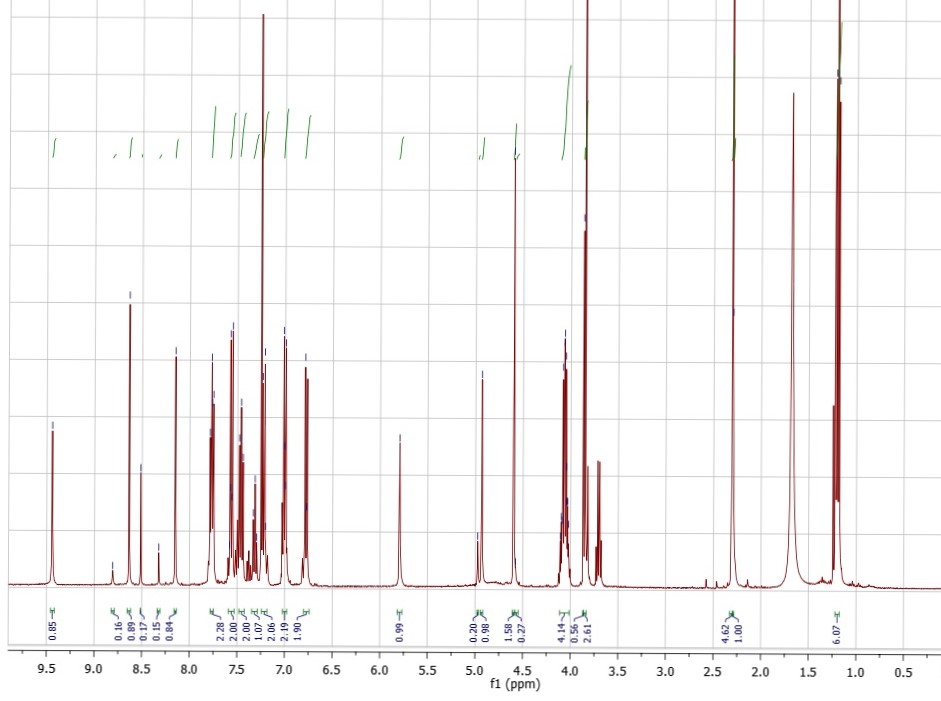


^13^C NMR spectrum of **5f**


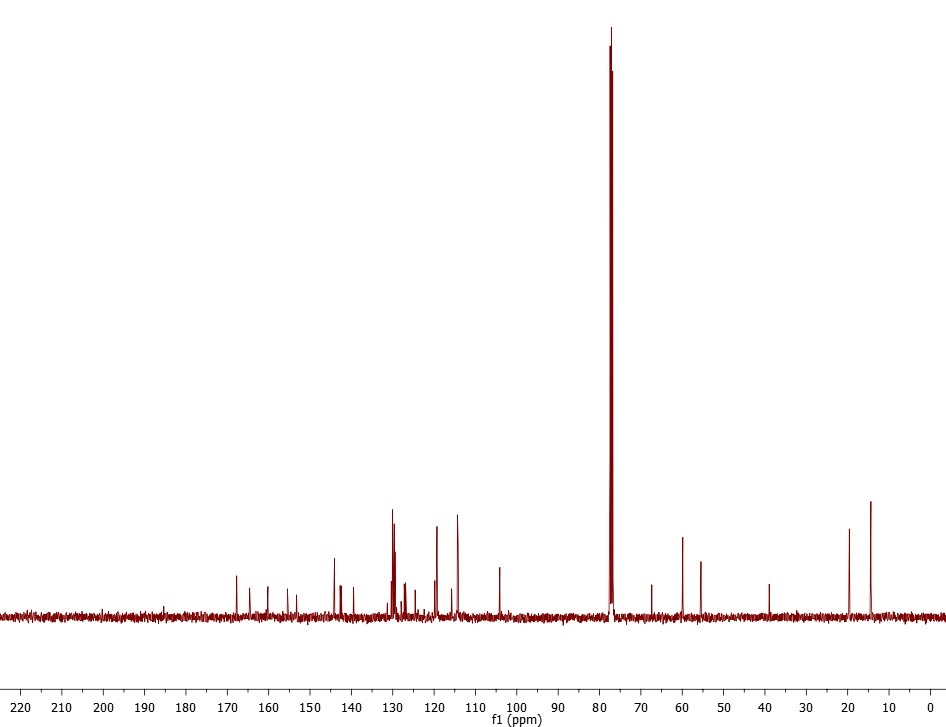


^1^H NMR spectrum of **5g**


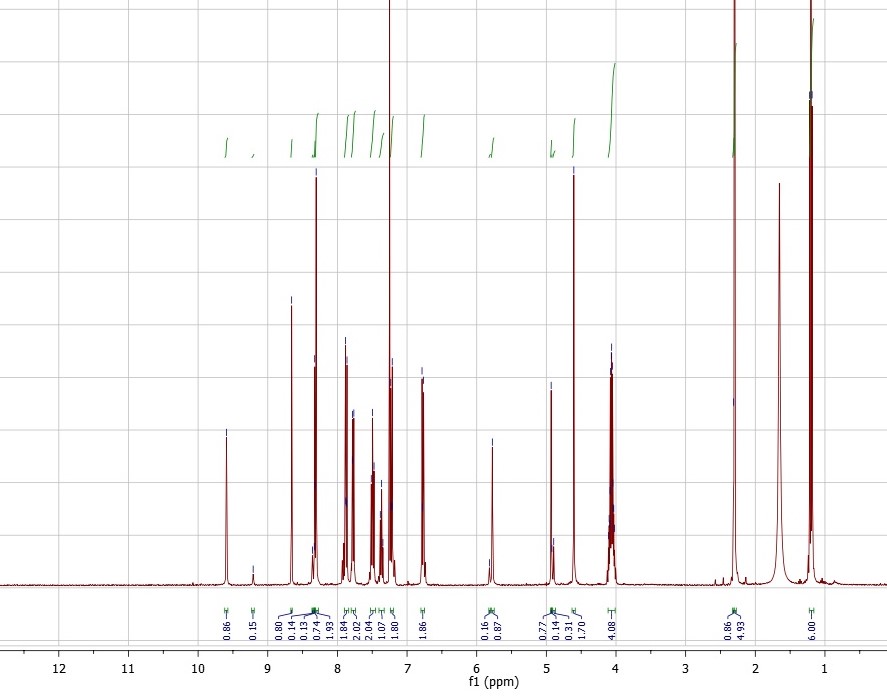


^13^C NMR spectrum of **5g**


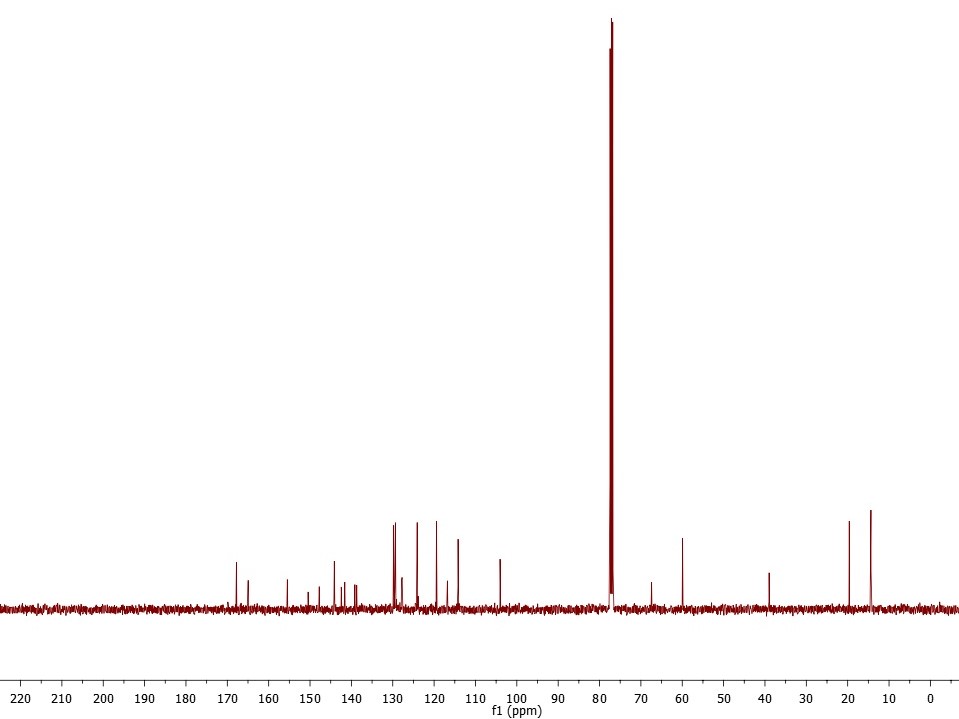


**References**

1. Ullooraa S, Shabarayab R, Ranganathanc R, Adhikari AV (2013) Synthesis, anticonvulsant and anti-inflammatory studies of new 1,4-dihydropyridin-4-yl phenoxy acetohydrazones. Eur J Med Chem 70:341-349
